# Historical Baseline Electrocardiogram Ascertainment Prior to Cancer Diagnosis and Cardiovascular Diagnosis Patterns in Cancer Survivors: A Retrospective Observational Cohort Study

**DOI:** 10.3390/cancers18203306

**Published:** 2026-10-14

**Authors:** Cameron Otto, Rushabh Shah, Sneha Nagavally, Kristen Osinski, Jeff Whittle, Sherry-Ann Brown

**Affiliations:** 1 Department of Medicine, Medical College of Wisconsin, Milwaukee, WI 53226, USA; 2 Clement J Zablocki VA Medical Center, Milwaukee, WI 53295, USA; 3 Department of Cardiovascular Medicine, Mayo Clinic, Rochester, MN 55905, USA; 4 Heart Innovation and Equity Research (HIER) Institute, Miami, FL 33131, USA

**Keywords:** cardio-oncology, cancer survivors, electrocardiography, ECG, cardiovascular disease, cardiovascular risk assessment, cardiovascular prevention, heart failure, atrial fibrillation, coronary artery disease, stroke, cardiotoxicity, cancer therapy, retrospective cohort, survivorship, artificial intelligence, AI-ECG, precision cardio-oncology, cardiovascular outcomes, cardiovascular screening

## Abstract

Background: Cardiovascular disease (CVD) remains a leading cause of morbidity and mortality among cancer survivors. Contemporary cardio-oncology frameworks emphasize baseline cardiovascular risk assessment before potentially cardiotoxic cancer therapies. Historical real world implementation of baseline electrocardiography (ECG), however, remains incompletely characterized. The objective of this study was to characterize historical baseline ECG ascertainment before cancer diagnosis and examine its association with documented cardiovascular diagnosis patterns in cancer survivors. Methods: We conducted a retrospective observational cohort study of 4592 adult cancer survivors within the Froedtert & Medical College of Wisconsin health system from 2000 to 2020. The analytic cohort was derived from a previously established longitudinal cancer survivor resource integrating demographic and cardiovascular clinical data, cancer treatment exposures, multimodality cardiac function ascertainment, and linked clinical, radiation oncology, and cancer registry information. Cardiac function ascertainment from an available cardiovascular imaging modality within three years before cancer diagnosis was one component of the analytic cohort definition. Patients were stratified by ascertainment of a 12-lead ECG within the same pre-diagnostic interval. Cardiovascular diagnoses documented before and after cancer diagnosis were characterized separately. For the newly documented diagnosis analyses, each cardiovascular outcome was evaluated among participants without a prior diagnosis of that same condition before cancer diagnosis. A multivariable logistic regression model evaluated the association between baseline ECG ascertainment and newly documented overall CVD diagnosis (including HF, AFIB, CAD, and stroke) while adjusting for age, sex, race, comorbidity count, and treatment count. Results: Among 4592 individuals, 3726 (81%) had a baseline ECG. ECG ascertainment was associated with older age and greater pre-existing cardiovascular and cardiometabolic burden. The available follow up distributions were similar between patients without and with baseline ECG ascertainment (748 [Q1 258, Q3 1781] vs. 767 [Q1 219, Q3 1822] days; *p* > 0.9). In multivariable analysis, baseline ECG ascertainment was associated with lower odds of a newly documented overall CVD diagnosis (OR 0.38, 95% CI 0.31–0.46); this odds ratio represents an association rather than a time-specific risk or protective effect. Conclusions: Baseline ECGs were commonly ascertainable within this longitudinal cancer survivor cohort and identified groups with different pre-existing cardiovascular burden and subsequent cardiovascular diagnosis patterns. These findings provide a historical benchmark for cardiovascular assessment in cancer care and support prospective patient-level studies integrating ECG findings with clinical and oncologic risk factors to refine cardiovascular surveillance strategies across cancer survivorship.

## Simple Summary

Cardiovascular disease is a major cause of illness and death among cancer survivors, making early cardiovascular assessment increasingly important. Electrocardiography (ECG) is an inexpensive, widely available, and noninvasive component of cardiovascular evaluation. In this retrospective study of 4592 adult cancer survivors, we characterized historical baseline ECG ascertainment during 2000–2020 and evaluated linkage with demographic characteristics, cancer treatment exposures, cardiovascular diagnoses, and longitudinal follow up. Most patients (81%) had a baseline ECG within three years before cancer diagnosis. Patients with baseline ECG ascertainment had greater documented cardiovascular and cardiometabolic burden before cancer diagnosis. We refined the results by separating descriptive cardiovascular diagnosis frequencies after cancer diagnosis from newly documented cardiovascular diagnoses among patients without the corresponding prior diagnosis. The observed associations are observational and hypothesis-generating.

## 1. Introduction

CVD is a leading non-malignant cause of morbidity and mortality among cancer survivors and represents a major competing risk to cancer-related outcomes [1,2,3]. As cancer survivorship continues to rise, now exceeding 17 million individuals in the United States and projected to surpass 22 million by 2030, the long-term health of this growing population has become a critical clinical and public health priority [1,2,3]. This elevated risk reflects a convergence of pre-existing cardiovascular risk factors, shared pathophysiologic mechanisms between cancer and CVD, cancer-treatment-related cardiotoxicity, and the underutilization of preventive cardiovascular care. Accordingly, an important question emerges regarding how baseline cardiovascular assessment was historically implemented across real world oncology populations prior to widespread maturation of modern cardio-oncology frameworks, and whether baseline ECG ascertainment during this period was associated with downstream cardiovascular outcomes in cancer survivors.

Many commonly used cancer therapies—including anthracyclines, HER2-targeted agents, and radiation—are inherently cardiotoxic and may induce a wide spectrum of cardiovascular complications, including arrhythmias, myocarditis, ischemia, and heart failure [4,5]. These risks have led to the emergence of cardio-oncology as a specialized field focused on the prevention, early detection, and management of cardiovascular disease in patients with cancer [6,7]. Contemporary cardio-oncology guidelines increasingly recommend comprehensive baseline cardiovascular risk assessment and optimization prior to potentially cardiotoxic therapies, including clinical history, physical examination, blood pressure assessment, laboratory evaluation, ECG, and selected imaging or biomarker strategies depending on baseline cardiovascular risk profile and anticipated treatment exposures [8,9,10]. However, substantial heterogeneity historically existed in real world implementation of baseline cardiovascular assessment across oncology populations, particularly during earlier eras preceding widespread cardio-oncology standardization. Clinicians thus continue to face challenges in identifying the highest risk patients. In addition, current guidelines lack specificity regarding ECG timing, frequency, and patient selection, leaving clinicians without a standardized protocol, and the absence of guideline uniformity, together with limited understanding of historical ECG ascertainment patterns and their associations with downstream cardiovascular outcomes in this population, impedes consistent implementation in clinical practice.

The electrocardiogram remains a cornerstone of cardiovascular assessment and is a low-cost, noninvasive, and widely accessible diagnostic modality capable of evaluating cardiac electrical activity [11]. By measuring the electrical impulses generated during cardiac contraction and relaxation, the ECG provides real-time data and insight into cardiac rhythm, conduction system integrity, ischemic changes, myocardial injury including myocardial infarction, and overall cardiac function [11]. Its strong diagnostic and monitoring potential has established it as a foundational tool in cardiology. Traditional ECG recordings provide only a snapshot of cardiac activity at a single point in time; however, advances in ambulatory ECG monitoring have extended its utility to continuous surveillance beyond the clinical setting [12]. In parallel, technological innovations, including artificial-intelligence-enhanced ECG interpretation, have expanded its role from diagnosis to prediction, enabling identification of subclinical dysfunction and estimation of future cardiovascular risk [13,14,15,16,17]. ECG screening has been explored in multiple populations, including individuals in higher-risk occupations, young athletes [18], and patients with pre-existing cardiovascular disease, and has been extended toward continuous and population-based screening strategies with the goal of diagnosing existing cardiovascular conditions and detecting potential future cardiac complications. In cancer populations, ECGs may be obtained for multiple reasons, including evaluation of pre-existing cardiovascular disease, assessment during cancer treatment planning, or for unrelated clinical encounters. A baseline ECG obtained prior to cancer diagnosis or treatment may therefore provide a more accurate representation of underlying cardiovascular substrate, independent of treatment-related effects, and may provide useful cardiovascular information prior to cancer diagnosis and treatment. Importantly, the present study was not designed to evaluate ECG as a substitute for comprehensive cardiovascular evaluation, nor to suggest that resting ECG alone is sufficient for the diagnosis or exclusion of cardiovascular disease in patients with cancer. Contemporary cardiovascular assessment appropriately incorporates clinical history, physical examination, laboratory evaluation, imaging, and additional testing when clinically indicated. Rather, this work evaluates historical real world ascertainment of baseline ECG prior to cancer diagnosis and treatment during a period spanning 2000–2020, before widespread implementation of contemporary cardio-oncology recommendations. ECG ascertainment therefore represents one measurable component of cardiovascular assessment within routine clinical practice and may reflect differences in cardiovascular awareness, surveillance intensity, referral behavior, or preventive care engagement. This population remains at elevated risk for developing cardiovascular complications, including heart failure, arrhythmias, and ischemic heart disease, due to the cardiotoxic effects of cancer therapies [5]. Within broader cardiovascular assessment strategies, ECG may contribute to earlier identification of cardiovascular abnormalities, risk stratification, and longitudinal surveillance. Yet, it remains unclear how historical baseline ECG ascertainment relates to subsequent cardiovascular diagnoses and newly documented cardiovascular diagnoses in cancer survivors. Furthermore, disparities in cardio-oncology care persist, with African American and other historically underserved populations experiencing disproportionate barriers to preventive cardiovascular care, including lower rates of screening, differences in access to care, and the effects of structural inequities and provider bias [19]. The distribution of baseline ECG utilization across sex and racial groups therefore remains important to evaluate.

In this retrospective observational study spanning 20 years of data, we characterize historical real world baseline ECG ascertainment before cancer diagnosis and examine its association with documented cardiovascular diagnoses within a previously established longitudinal cancer survivor cohort. The cohort integrates demographic and cardiovascular clinical data; cancer treatment exposures; multimodality cardiac function ascertainment; and linked clinical, radiation oncology, and cancer registry information.

## 2. Methods

### 2.1. Study Design and Setting

We conducted a retrospective cohort study designed to investigate the relationship between baseline ECG acquisition and the subsequent development of CVD among cancer survivors. This work draws upon a pre-established, racially and clinically diverse cohort of adult cancer survivors derived from the Froedtert & Medical College of Wisconsin (MCW) clinical data research warehouse (CRDW), an academic health system serving a racially diverse urban population. The study period intentionally spanned two decades (2000–2020) in order to characterize historical real world cardiovascular assessment practices across oncology populations during eras preceding and overlapping the emergence of contemporary cardio-oncology frameworks.

The study was constructed to characterize historical baseline ECG ascertainment prior to cancer diagnosis and its relationship with downstream cardiovascular diagnoses across cancer populations, demographic subgroups, and cancer treatment exposures. We analyzed ECGs obtained before cancer diagnosis rather than only before cancer treatment in order to identify a pre-diagnostic cardiovascular baseline. The study evaluated ECG ascertainment within routine clinical practice and the availability of linked longitudinal clinical data. Because the cohort had been previously characterized, demographic characteristics, cardiovascular diagnoses, and treatment exposures were available for focused analysis.

We specifically chose to investigate ECGs obtained before cancer diagnosis and not just before cancer treatment. When deciding a treatment plan, an ECG may be obtained for immediate therapeutic decision-making; however, this was not the relationship we aimed to analyze. By restricting the analysis to ECGs obtained prior to diagnosis, we avoided conflating diagnostic ECGs obtained for immediate therapeutic decision-making with baseline ECGs reflecting underlying cardiovascular risk. This design enabled us to evaluate associations between baseline ECG ascertainment and downstream cardiovascular outcomes independent of immediate treatment-planning ECG utilization.

Cardiovascular diagnoses documented before and after cancer diagnosis were tabulated separately to preserve temporal context. The post-cancer comparisons used the full baseline ECG and no baseline ECG cohorts and therefore represent observed cardiovascular diagnosis frequencies after cancer diagnosis rather than disease-free incident rates. Given well documented disparities in cardiovascular care, we also examined differences in ECG ascertainment and cardiovascular diagnoses across race and sex.

An additional objective was to evaluate whether historical ECG data were ascertainable at sufficient scale and linkable with longitudinal cardiovascular outcomes, demographic diversity, and treatment heterogeneity to support subsequent endpoint-specific statistical investigations.

Institutional Review Board approval was obtained for this retrospective study, and informed consent was waived.

### 2.2. Study Population

**Figure 1:**
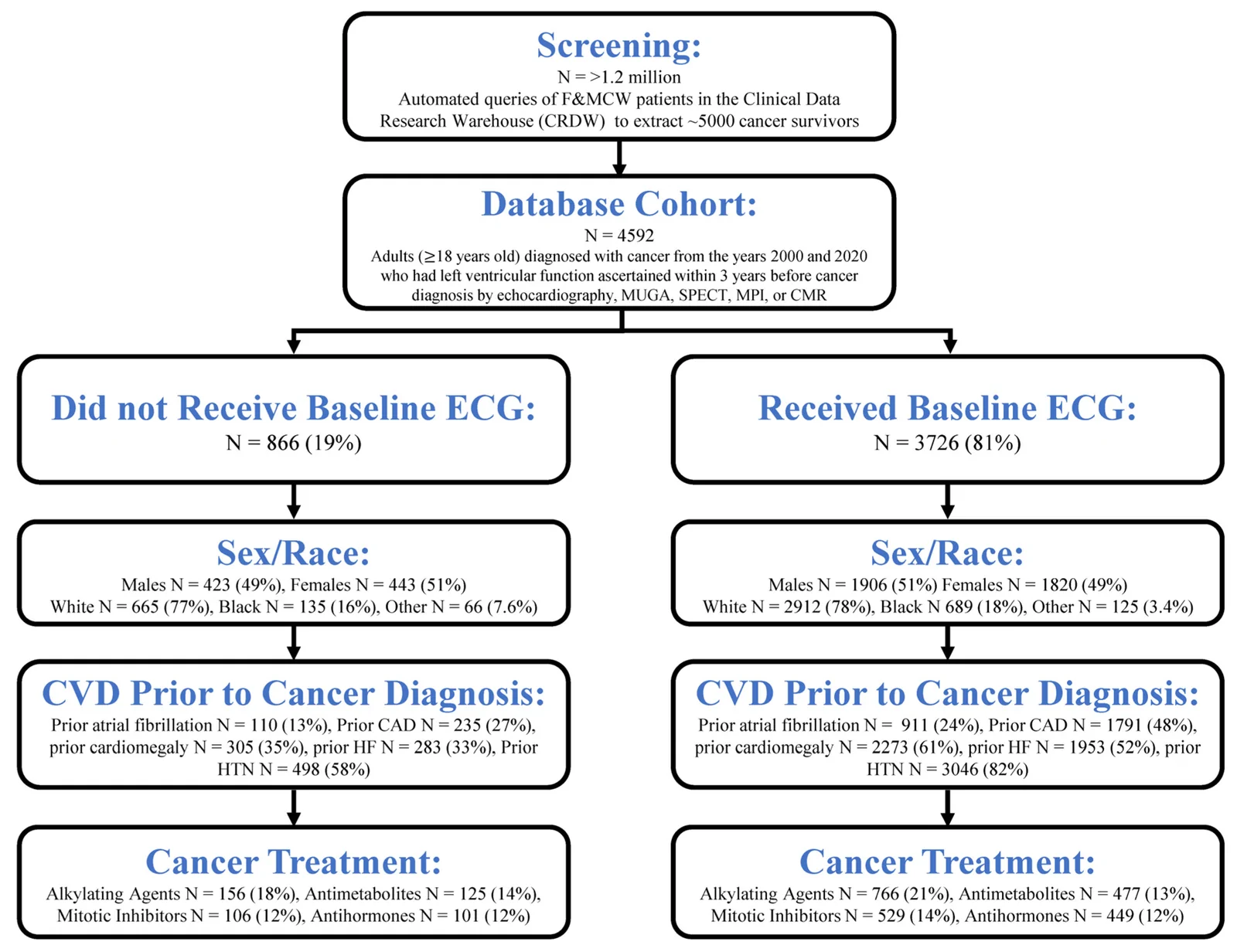
Development of the study cohort. Cohort construction integrated adult cancer diagnosis, demographic and cardiovascular clinical data, cancer treatment exposures, multimodality cardiac function ascertainment, and linked clinical data sources. Cardiac function ascertainment from echocardiography, MUGA, SPECT MPI, or CMR within three years before cancer diagnosis was one component of the analytic cohort definition.

The analytic cohort comprised 4592 adult patients (≥18 years of age) diagnosed with cancer between 2000 and 2020 and was derived from a previously established longitudinal cancer survivor resource integrating demographic and cardiovascular clinical data, cancer diagnosis and treatment exposures, multimodality cardiac function ascertainment, and linked clinical data sources. As one component of the analytic cohort definition, left ventricular ejection fraction or cardiac function was ascertainable from an available cardiovascular imaging modality, including echocardiography, MUGA, SPECT MPI, or CMR, within three years prior to cancer diagnosis (Figure 1).

The dataset was built from the Froedtert & MCW CRDW using the Informatics for Integrating Biology & the Bedside (I2B2) toolset. Patients were stratified based on whether they received a standard 12-lead ECG within the same three-year pre-diagnostic interval. The CRDW/I2B2 cohort data were supplemented with radiation oncology information from the Mosaic database and cancer registry information from SEER. The multimodal data linkage architecture is summarized in Supplemental Figure S1.

### 2.3. Data Elements and Definitions

Demographic, epidemiologic, cardiovascular outcome, cancer diagnosis, cancer treatment, and baseline ECG data were compiled for analysis. Extracted data included demographic variables (age, sex, race), clinical history of cardiovascular disease and associated risk factors, baseline ECG receipt, cancer diagnosis, and cancer treatment exposures. Cancer treatment exposure was characterized in detail by pharmacologic class, and the specific antineoplastic agents represented within each class are provided in Supplemental Table S1. The dataset incorporated a broad range of clinical variables relevant to cardiovascular risk and outcomes. Detailed longitudinal data regarding physical examination findings, stress testing, downstream imaging indications, biomarker evaluation, initiation and duration of cardiovascular or cardioprotective medications, interventional procedures, referral patterns, and longitudinal cardiovascular management decisions were not uniformly available in structured form across the retrospective study period.

Baseline ECG was defined as the presence of a 12-lead ECG performed within three years prior to cancer diagnosis. The study evaluated ascertainment of ECG within routine clinical practice and did not evaluate ECG findings as isolated diagnostic determinants of cardiovascular disease. Cardiovascular disease before and after cancer diagnosis was analyzed separately.

Cancer treatments were classified using Medispan pharmacologic categories, including alkylating agents, antimetabolites, angiogenesis inhibitors, HER2 antibodies, antineoplastic antibodies, antineoplastic hormonal drugs, antineoplastic antibiotics (including anthracyclines), combined antineoplastics, antineoplastic enzyme inhibitors, miscellaneous antineoplastics, chemotherapy rescue drugs, immune agents, and mitotic inhibitors. Specific drugs corresponding to each category are listed in Supplemental Table S1, which directly reflects the Medispan classification framework.

### 2.4. Cardiovascular Outcomes and Risk Factor Ascertainment

Cardiovascular outcomes and comorbidities—including HF, AFIB, MI, coronary artery disease (CAD), stroke, cardiomyopathy, restrictive cardiomyopathy, cardiomegaly, cerebral atherosclerosis, peripheral artery disease (PAD), unspecified peripheral vascular disease (UPVD), diabetes, hypertension, hyperlipidemia, orthopnea, dyspnea, shortness of breath, and fatigue—were extracted using International Classification of Diseases, Ninth and Tenth Revision (ICD-9 and ICD-10) diagnosis codes. Both inpatient and outpatient encounters were included, and all codes were manually reviewed and validated to ensure consistent phenotyping.

Cardiovascular diagnoses documented before and after cancer diagnosis were analyzed separately using structured ICD-9 and ICD-10 diagnosis codes. Descriptive post-cancer diagnosis frequencies were summarized using the observed yes/no denominator for each outcome within the baseline ECG and no baseline ECG groups; outcome-specific denominators and NMiss counts from the analysis output are reported in Supplemental Table S2. For the newly documented diagnosis analyses, each cardiovascular outcome was evaluated among participants without a prior diagnosis of that same condition before cancer diagnosis. The denominator therefore comprised participants without the corresponding prior diagnosis, and the numerator comprised those subsequently diagnosed with that condition after cancer diagnosis.

Overall CVD was an established recorded cardiovascular diagnosis variable in the cohort and was analyzed separately from the individually assessed HF, AFIB, CAD, MI, and stroke outcomes. The available data do not define overall CVD as a validated time to first event composite with adjudicated component membership. Accordingly, the manuscript reports the overall CVD result alongside each individual cardiovascular diagnosis, with outcome-specific denominators and missing counts provided separately.

### 2.5. Endpoints

The primary outcome was a newly documented overall CVD diagnosis after cancer diagnosis among participants without a prior documented overall CVD diagnosis, compared according to baseline ECG ascertainment status.

Secondary outcomes were newly documented HF, AFIB, CAD, MI, and stroke after cancer diagnosis among participants without the corresponding prior documented cardiovascular diagnosis.

Exploratory endpoints included cardiovascular diagnosis frequencies across cancer treatment classes; differences in ECG ascertainment and cardiovascular diagnoses across sex and racial subgroups; available follow up; and an adjusted model evaluating newly documented overall CVD diagnosis. Cancer-specific survival or tumor survival was not an endpoint of the current study.

### 2.6. Statistical Analysis

Descriptive statistics were generated by baseline ECG status. Continuous variables were summarized as mean +/− standard deviation and compared using Student’s *t*-tests. Categorical variables were summarized as counts and percentages and compared using chi-square tests. Descriptive post-cancer diagnosis percentages used the observed yes/no denominator for the corresponding outcome. For the newly documented diagnosis analyses, the numerator was dn_* = yes and the denominator was the corresponding population with pr_* = no. The six newly documented diagnosis comparisons were evaluated using chi-square tests and adjusted using the Benjamini–Hochberg false discovery rate (FDR) method. Available follow up duration (Survdays) was summarized as median and interquartile range and compared between ECG groups using the Wilcoxon rank-sum test.

For the primary newly documented diagnosis comparisons, we report event counts, denominators, absolute differences in documented diagnosis proportions, and 95% confidence intervals using the Newcombe method combining Wilson score limits without continuity correction [20]. The six newly documented diagnosis comparisons were evaluated using chi-square tests and adjusted using the Benjamini–Hochberg false discovery rate (FDR) method. *p* values for other tabulations are retained from the original analysis. Sparse treatment subgroup comparisons are interpreted descriptively, and when neither group has an event, no inferential comparison is reported.

Cardiovascular outcomes (e.g., composite CVD, HF, AFIB, CAD, MI, and stroke) were compared across patients who were prescribed various cancer treatments. These comparisons were first performed across treatment classes and then specifically across patients receiving individual treatment categories. Following these comparisons, cardiovascular outcomes were evaluated within sex groups among patients receiving specific treatments and then within racial groups (White, Black, and Other) among patients receiving those same treatments, thereby preserving the nested analytical structure of treatment-specific subgroup comparisons.

Multivariable logistic regression was used to assess predictors of baseline ECG ascertainment, including race and sex. In response to the marked baseline clinical imbalance between ECG groups, an exploratory multivariable logistic regression model evaluating newly documented overall CVD diagnosis was also evaluated with baseline ECG status, age, sex, race, comorbidity count, and treatment count as covariates. Odds ratios (ORs) and 95% confidence intervals were reported. The model is interpreted as an adjusted observational association rather than a causal effect estimate.

The baseline clinical burden includes pre-existing cardiovascular diseases and cardiometabolic conditions including diabetes, hypertension, and hyperlipidemia. A simple comorbidity count does not preserve the identity, severity, duration, control, or combinations of individual conditions, and residual confounding may remain. A separate exploratory model evaluated baseline ECG ascertainment as the exposure of interest and newly documented overall CVD diagnosis after cancer diagnosis as the outcome, with adjustment for age, sex, race, comorbidity count, and treatment count. The model estimates odds of a documented diagnosis and does not estimate time-specific cardiovascular risk, hazard, cumulative incidence, risk reduction, prevention, or a protective effect of ECG ascertainment.

To account for multiple comparisons, *p* values were adjusted using the Benjamini–Hochberg false discovery rate (FDR) method. Sparse subgroup findings were interpreted using event counts and effect estimates and were treated as exploratory rather than as definitive negative findings.

All statistical analyses were conducted using R version 4.1.3, with required packages installed and loaded as needed.

### 2.7. Data Analysis Strategy

The analytical framework was aligned with the study objectives. Analyses began with characterization of the cohort and baseline ECG distribution, followed by evaluation of cardiovascular diagnoses documented before and after cancer diagnosis. We then refined the results by evaluating newly documented overall CVD and individual cardiovascular diagnoses among participants without the corresponding prior documented diagnosis. Available follow up was compared between ECG groups, and an exploratory adjusted model evaluated newly documented overall CVD diagnosis. Subsequent analyses examined treatment classes and demographic subgroups. The observational comparisons characterize the assembled longitudinal resource and generate hypotheses for subsequent endpoint validated time to event analyses. Sparse treatment subgroup comparisons are descriptive and exploratory; comparisons with no events in both groups are not subjected to an inferential test.

## 3. Results

### 3.1. Cohort Characteristics

We analyzed a cohort of 4592 cancer survivors, of whom 3726 (81%) received a baseline ECG and 866 (19%) did not receive a baseline ECG (Figure 1). This cohort reflects real world cardiovascular assessment practices across a 20-year period spanning eras before and during the early maturation of contemporary cardio-oncology frameworks. The mean age of patients with baseline ECG was 69.17 ± 12.87 years compared with 66.94 ± 14.10 years in those without ECG (*p* < 0.001).

Baseline ECGs were ascertainable for 81% of this large real world cohort, demonstrating substantial historical ECG availability for linkage with demographic, treatment, and longitudinal cardiovascular data. There was no significant difference in sex distribution between groups (female: 1820 [49%] with ECG vs. 443 [51%] without; male: 1906 [51%] vs. 423 [49%], *p* = 0.4). Similarly, there was no significant difference in race between Black and White patients (White: 2912 [78%] vs. 665 [77%]; Black: 689 [18%] vs. 135 [16%], *p* = 0.14). Patients categorized as Other (Asian, Pacific Islander, Native American) were less likely to have baseline ECG ascertainment (125 [3.4%] vs. 66 [7.6%], *p* < 0.001). Demographic data are summarized in Table 1.

### 3.2. Cardiovascular Disease and Risk Factors Prior to Cancer Diagnosis

Patients undergoing baseline ECG ascertainment demonstrated substantially greater baseline cardiovascular disease burden and cardiometabolic risk factor prevalence prior to cancer diagnosis. Composite CVD was previously diagnosed in 1952 of 3726 patients (52%) with baseline ECG compared to 322 of 866 patients (37%) without (*p* < 0.001).

**Figure 2:**
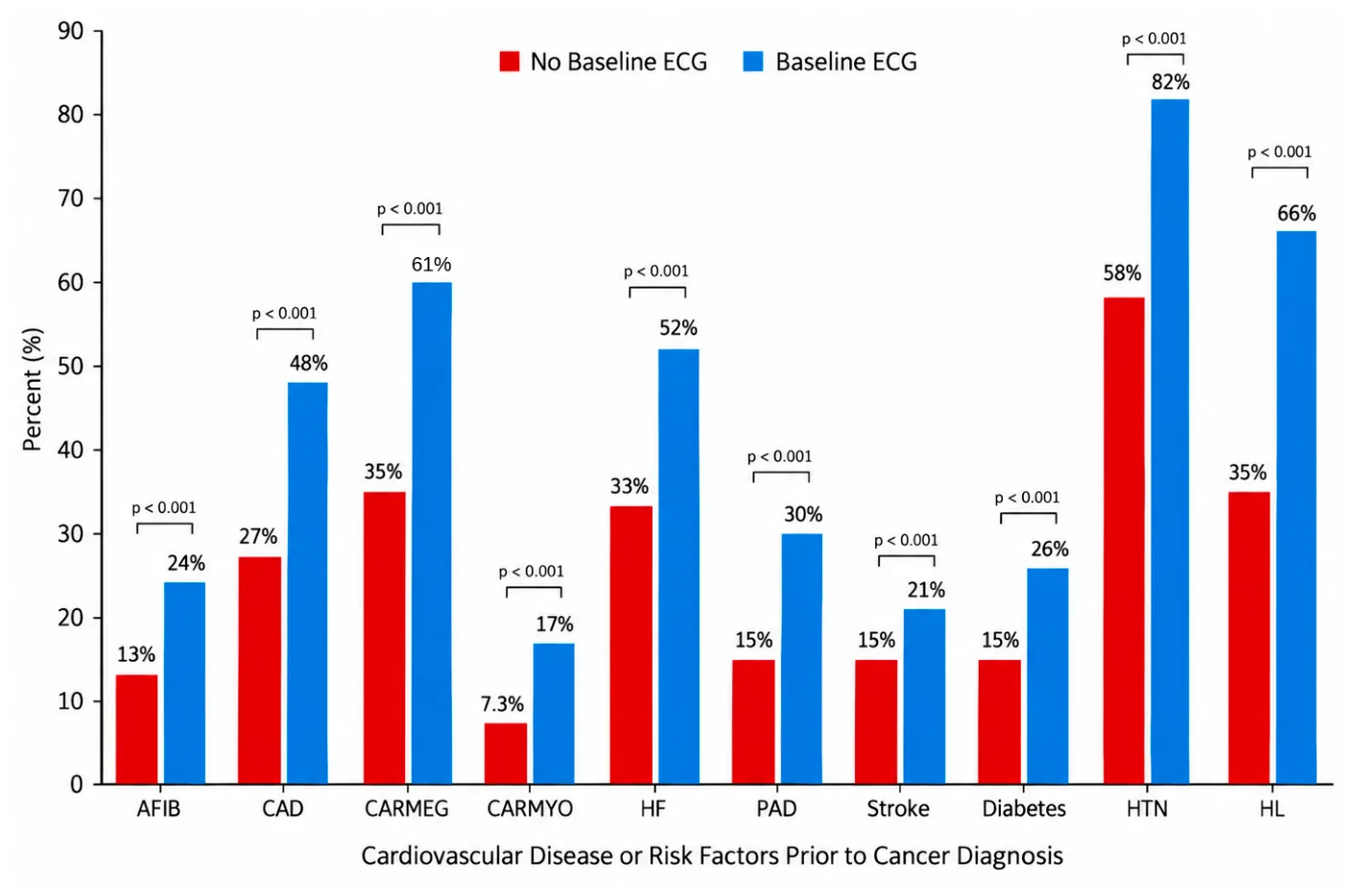
Cardiovascular disease or risk factors prior to cancer diagnosis according to baseline ECG ascertainment. AFIB = atrial fibrillation, CAD = coronary artery disease, CARMEG = cardiomegaly, CARMYO = cardiomyopathy, HF = heart failure, PAD = peripheral artery disease, HTN = hypertension, HL = hyperlipidemia. The *y* axis presents prevalence within each ECG ascertainment group. *p* values compare the baseline ECG and no baseline ECG groups.

Specific cardiovascular conditions prior to cancer diagnosis included AFIB (911 [24%] vs. 110 [13%], *p* < 0.001), HF (1953 [52%] vs. 283 [33%], *p* < 0.001), CAD (1791 [48%] vs. 235 [27%], *p* < 0.001), cardiomegaly (2273 [61%] vs. 305 [35%], *p* < 0.001), cardiomyopathy (621 [17%] vs. 63 [7.3%], *p* < 0.001), restrictive cardiomyopathy (398 [11%] vs. 57 [6.6%], *p* < 0.001), PAD (1128 [30%] vs. 128 [15%], *p* < 0.001), and stroke (789 [21%] vs. 127 [15%], *p* < 0.001) (Figure 2). Additional comorbidities included cerebral atherosclerosis (67 [1.8%] vs. five [0.6%], *p* = 0.029) and unspecified peripheral vascular disease (UPVD: 576 [15%] vs. 70 [8.1%], *p* < 0.001).

Cardiovascular risk factors included MI (357 [9.6%] vs. 51 [5.9%], *p* = 0.002), diabetes (953 [26%] vs. 131 [15%], *p* < 0.001), hypertension (3046 [82%] vs. 498 [58%], *p* < 0.001), and hyperlipidemia (2468 [66%] vs. 305 [35%], *p* < 0.001).

We also characterized additional diagnoses, clinical signs, and symptoms documented before cancer diagnosis. Peripheral edema was noted in 1265 (34%) vs. 164 (19%) (*p* < 0.001), orthopnea in 63 (1.7%) vs. four (0.5%) (*p* = 0.022), shortness of breath in 2257 (61%) vs. 366 (42%) (*p* < 0.001), and fatigue in 1808 (49%) vs. 227 (26%) (*p* < 0.001). Epidemiologic data are summarized in Table 2. These findings indicate that baseline ECG ascertainment occurred more frequently among individuals with greater documented cardiovascular and cardiometabolic burden within routine clinical practice.

### 3.3. Cardiovascular Outcomes After Cancer Diagnosis

Following cancer diagnosis, the frequencies of composite CVD and individually assessed HF, AFIB, CAD, MI, and stroke diagnoses were lower among patients with baseline ECG ascertainment than among those without baseline ECG ascertainment. These comparisons describe diagnosis frequencies in the available clinical record after cancer diagnosis and do not establish causal effects of ECG testing.

**Figure 3:**
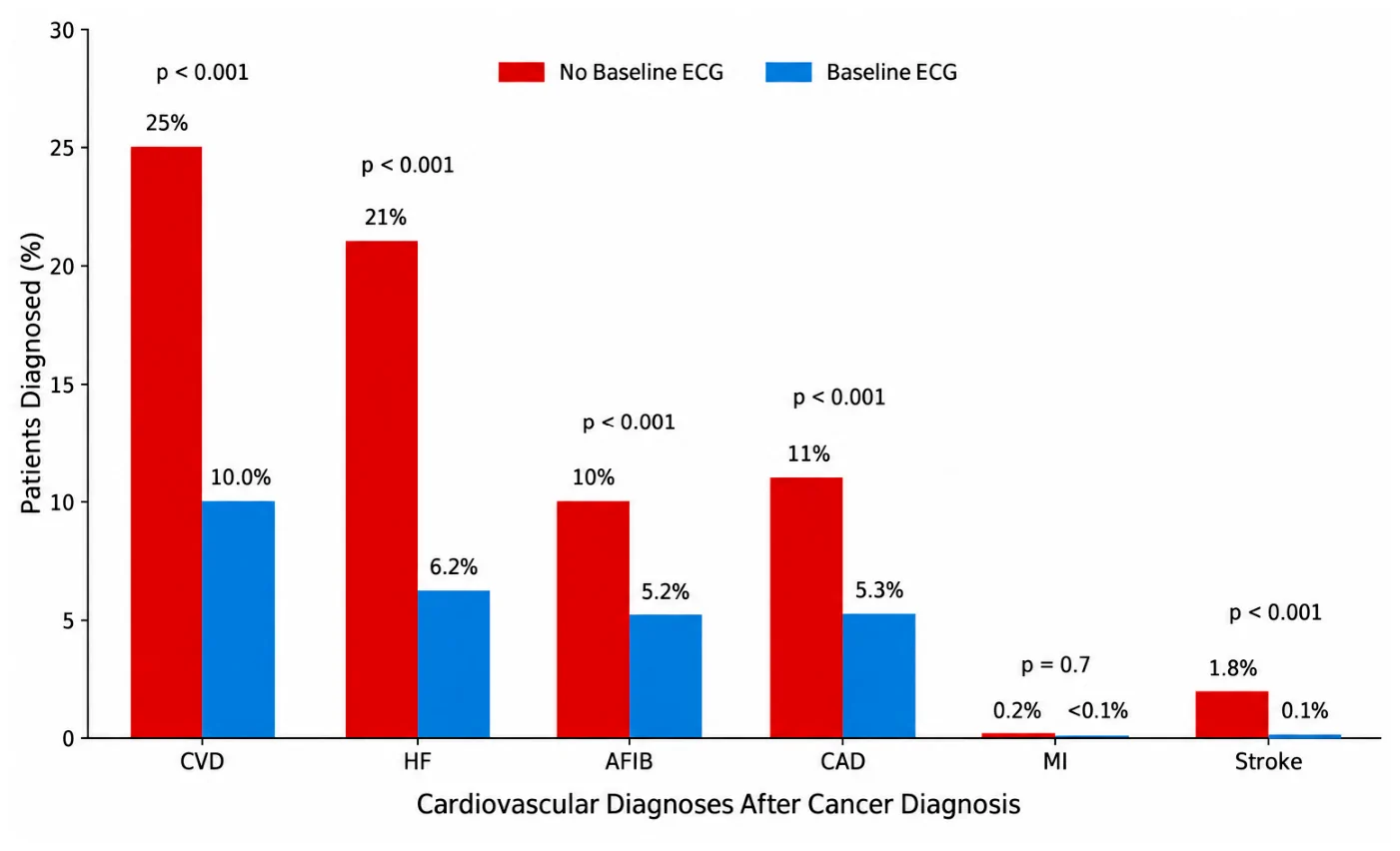
The frequency of cardiovascular diagnoses after cancer diagnosis according to baseline ECG ascertainment. HF = heart failure, AFIB = atrial fibrillation, CAD = coronary artery disease, MI = myocardial infarction. Percentages represent diagnosis frequencies within the baseline ECG and no baseline ECG groups. Outcome-specific denominators and NMiss counts are provided in Supplemental Table S2.

After cancer diagnosis, composite CVD was diagnosed in 372 of 3726 patients (10.0%) with baseline ECG ascertainment compared with 213 of 866 (25%) without (*p* < 0.001). HF was diagnosed in 137 (6.2%) vs. 123 (21%) (*p* < 0.001), AFIB in 157 (5.2%) vs. 77 (10%) (*p* < 0.001), CAD in 129 (5.3%) vs. 74 (11%) (*p* < 0.001), and stroke in five (0.1%) vs. 15 (1.8%) (*p* < 0.001) (Figure 3). Myocardial infarction was rare (three [<0.1%] vs. two [0.2%], *p* = 0.7).

Despite greater documented cardiovascular and cardiometabolic burden before cancer diagnosis, patients with baseline ECG ascertainment had lower frequencies of cardiovascular diagnoses after cancer diagnosis. The retrospective design, baseline group differences, clinical indications for ECG testing, and available follow up preclude causal interpretation. These findings are therefore treated as observational and hypothesis-generating.

### 3.4. Newly Documented Cardiovascular Diagnoses After Cancer Diagnosis

To distinguish descriptive post-cancer diagnosis frequencies from newly documented diagnoses among patients without the corresponding prior diagnosis, we refined the results by evaluating each cardiovascular outcome among participants without that documented cardiovascular diagnosis before cancer diagnosis. These analyses identify newly documented diagnoses in the available clinical record and do not establish adjudicated incident events within a validated time to event framework.

**Figure 4:**
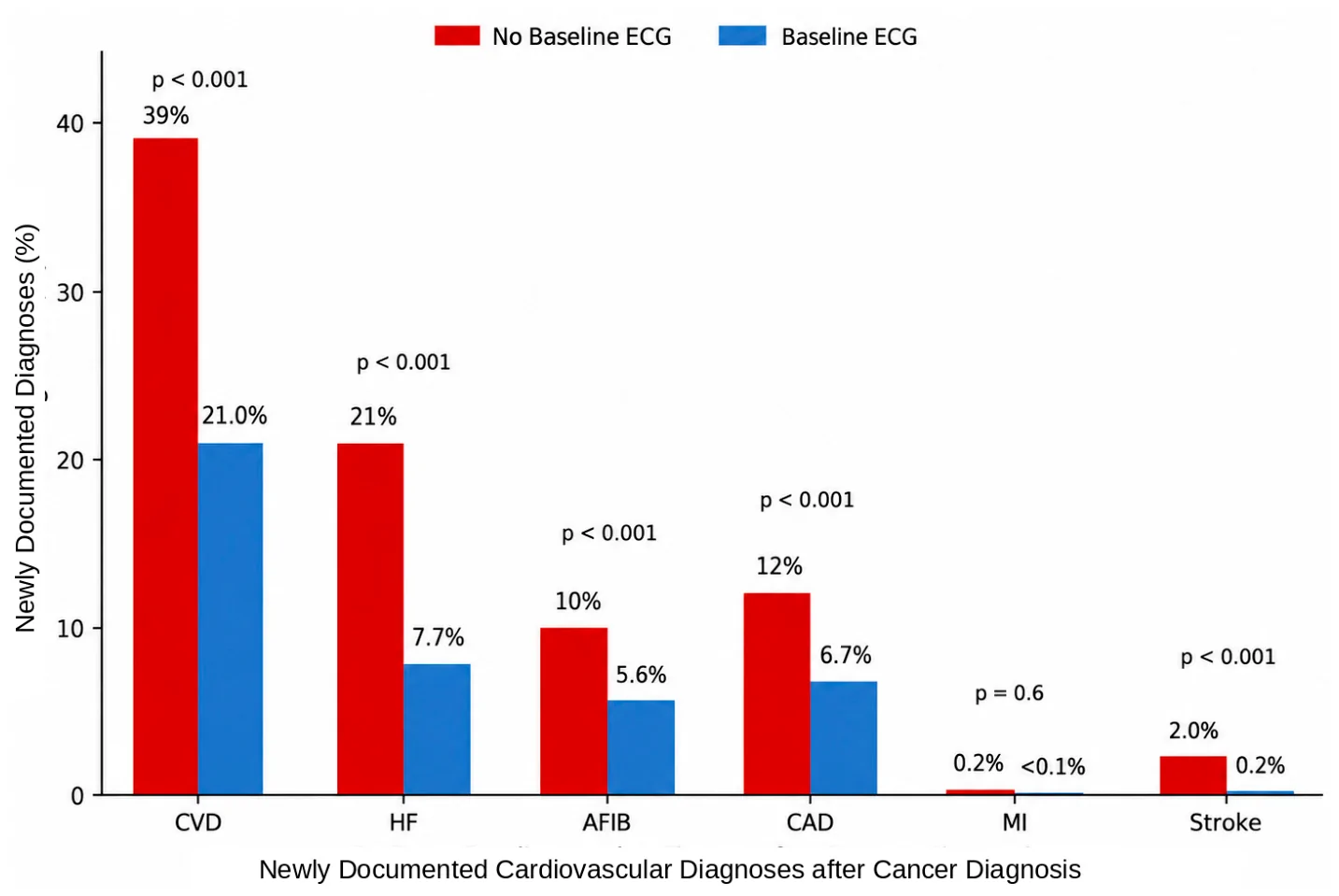
Newly documented cardiovascular diagnoses after cancer diagnosis according to baseline ECG ascertainment. These are diagnosis-based comparisons, not validated time to event incidence estimates.

Newly documented overall CVD diagnosis occurred in 372 of 1774 patients (21.0%) with baseline ECG ascertainment compared with 213 of 544 (39%) without baseline ECG ascertainment (FDR-adjusted *p* < 0.001) (Table 3). Newly documented HF occurred in 137 of 1773 (7.7%) vs. 123 of 583 (21%), AFIB in 157 of 2815 (5.6%) vs. 77 of 756 (10%), CAD in 129 of 1935 (6.7%) vs. 74 of 631 (12%), MI in three of 3369 (<0.1%) vs. two of 815 (0.2%), and stroke in five of 2937 (0.2%) vs. 15 of 739 (2.0%), respectively. FDR-adjusted *p* values were <0.001 for HF, AFIB, CAD, and stroke and 0.6 for MI. Diagnoses are summarized in Figure 4. 

**Table 3:** Newly documented cardiovascular diagnoses after cancer diagnosis among patients without the corresponding prior documented diagnosis, according to baseline ECG ascertainment.

Outcome	No Baseline ECG n/N (%)	Baseline ECG n/N (%)	FDR-Adjusted *p* Value
Overall CVD	213/544 (39%)	372/1774 (21.0%)	<0.001
HF	123/583 (21%)	137/1773 (7.7%)	<0.001
AFIB	77/756 (10%)	157/2815 (5.6%)	<0.001
CAD	74/631 (12%)	129/1935 (6.7%)	<0.001
MI	2/815 (0.2%)	3/3369 (<0.1%)	0.6
Stroke	15/739 (2.0%)	5/2937 (0.2%)	<0.001

For each outcome, the denominator is the number of patients without that cardiovascular diagnosis before cancer diagnosis (pr_* = no), and the numerator is the corresponding newly documented diagnosis after cancer diagnosis (dn_* = yes). *p* values are Benjamini–Hochberg FDR-adjusted across the six newly documented diagnosis comparisons. Overall CVD combines HF, AFIB, CAD, and stroke.

### 3.5. Follow Up Duration and Exploratory Adjusted Analysis

Median available follow up was 748 days (Q1 258, Q3 1781) without baseline ECG ascertainment and 767 days (Q1 219, Q3 1822) with baseline ECG ascertainment (Wilcoxon rank-sum *p* > 0.9) (Table 4). Follow up was unavailable for 63 and 308 patients, respectively. This comparison is descriptive and does not account for censoring, time to first documented diagnosis, loss to follow up, endpoint-specific observation time, or competing risk of death; it therefore does not establish equivalent follow up opportunities between groups.

In the exploratory multivariable logistic regression model for newly documented overall CVD diagnosis after cancer diagnosis, baseline ECG ascertainment was associated with lower odds of the documented diagnosis (OR 0.38, 95% CI 0.31–0.46; *p* < 0.001) after adjustment for age, sex, race, comorbidity count, and treatment count (Table 5). The odds ratio is an adjusted observational association and does not estimate time-specific cardiovascular risk, hazard, cumulative incidence, risk reduction, prevention, or a protective effect of ECG ascertainment. Residual confounding remains possible.

**Figure 5:**
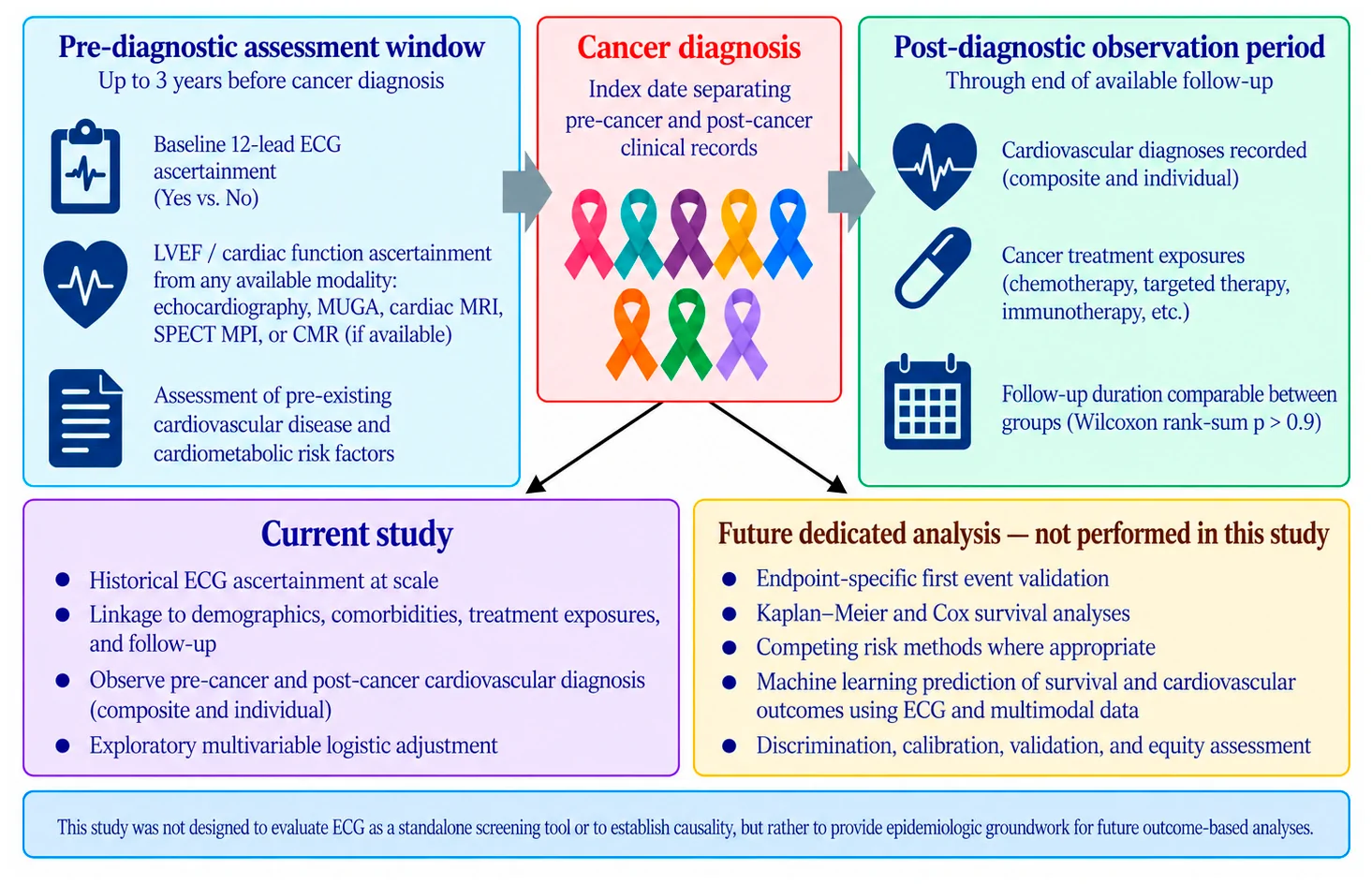
The study design, data linkage, and interpretation framework. The figure summarizes the pre-diagnostic assessment window, baseline ECG ascertainment, multimodality cardiac function ascertainment, the cancer diagnosis index date, post-diagnostic observation, and the analyses completed in the current study. It distinguishes the current study from endpoint-specific first event validation, Kaplan–Meier and Cox survival analysis, competing risk methods, and machine learning prediction reserved for a subsequent dedicated study.

Figure 5 summarizes the temporal organization, linked data domains, and analytic boundaries of the current study. The pre-diagnosis assessment window extended up to three years before cancer diagnosis and included ascertainment of a baseline 12-lead ECG. For the current ECG analytic cohort, left ventricular ejection fraction or cardiac function was ascertained from available cardiovascular imaging modalities, including echocardiography, MUGA, SPECT MPI, and CMR, within three years before cancer diagnosis. Cancer diagnosis served as the index date separating the pre-cancer and post-cancer clinical record periods. Following cancer diagnosis, the available longitudinal record was used to characterize cardiovascular diagnoses, cancer treatment exposures, and available follow up. Within the current study, these linked data were used to characterize historical ECG ascertainment, demographic and comorbidity patterns, treatment exposures, descriptive post-cancer diagnosis frequencies, newly documented diagnoses among patients without the corresponding prior documented diagnosis, and an exploratory multivariable logistic model. Endpoint-specific first event validation, Kaplan–Meier and Cox survival analyses, competing risk methods where appropriate, and artificial intelligence prediction of survival and cardiovascular outcomes are reserved for a subsequent dedicated study. The linked data source framework includes the Froedtert & MCW CRDW queried through I2B2, Mosaic radiation oncology data, and SEER cancer registry information.

### 3.6. Cancer Treatment Exposure by Baseline ECG Status

We analyzed the types of cancer treatments received by patients with and without baseline ECG. There was no significant difference in the proportion of patients treated with alkylating agents (21% vs. 18%, *p* = 0.2), antimetabolites (13% vs. 14%, *p* = 0.4), HER2 antibodies (1.3% vs. 0.8%, *p* = 0.5), antineoplastic hormonal drugs (12% vs. 12%, *p* = 0.9), combined antineoplastics (0.7% vs. 0.3%, *p* = 0.5), antineoplastic enzyme inhibitors (5.4% vs. 5.1%, *p* = 0.9), miscellaneous antineoplastics (3.0% vs. 4.2%, *p* = 0.2), chemotherapy rescue drugs (4.9% vs. 5.0%, *p* > 0.9), or mitotic inhibitors (14% vs. 12%, *p* = 0.2).

However, significant differences were observed for angiogenesis inhibitors (2.7% vs. 1.3%, *p* = 0.044), antineoplastic antibodies (9.3% vs. 6.4%, *p* = 0.017), antineoplastic antibiotics (9.4% vs. 12%, *p* = 0.021), and immune agents (3.2% vs. 6.1%, *p* < 0.001). Further information regarding treatment exposure is provided in Table 6.

**Table 6:** Cancer treatment data of individuals based on receipt of baseline ECG.

Drug Class	Treatment	No Baseline ECG	Baseline ECG	*p* Value
Alkylating Agents	Not treated with	710 (82%)	2960 (79%)	0.2
	Treated with	156 (18%)	766 (21%)	
Antimetabolites	Not treated with	741 (86%)	3249 (87%)	0.4
	Treated with	125 (14%)	477 (13%)	
Angiogenesis Inhibitors	Not treated with	855 (99%)	3627 (97%)	0.044
	Treated with	11 (1.3%)	99 (2.7%)	
Anti HER2 Antibodies	Not treated with	859 (99%)	3678 (99%)	0.5
	Treated with	7 (0.8%)	48 (1.3%)	
Antineoplastic Antibodies	Not treated with	811 (94%)	3381 (91%)	0.017
	Treated with	55 (6.4%)	345 (9.3%)	
Antineoplastic Hormones	Not treated with	765 (88%)	3277 (88%)	0.9
	Treated with	101 (12%)	449 (12%)	
Antineoplastic Antibiotics	Not treated with	759 (88%)	3377 (91%)	0.021
	Treated with	107 (12%)	349 (9.4%)	
Combined Antineoplastics	Not treated with	863 (100%)	3701 (99%)	0.5
	Treated with	3 (0.3%)	25 (0.7%)	
Antineoplastic Enzymes	Not treated with	822 (95%)	3525 (95%)	0.9
	Treated with	44 (5.1%)	201 (5.4%)	
Miscellaneous Antineoplastics	Not treated with	830 (96%)	3616 (97%)	0.2
	Treated with	36 (4.2%)	110 (3.0%)	
Chemotherapy Rescue Drugs	Not treated with	823 (95%)	3545 (95%)	>0.9
	Treated with	43 (5.0%)	181 (4.9%)	
Immune Agents	Not treated with	813 (94%)	3608 (97%)	<0.001
	Treated with	53 (6.1%)	118 (3.2%)	
Mitotic Inhibitors	Not treated with	760 (88%)	3197 (86%)	0.2
	Treated with	106 (12%)	529 (14%)	

### 3.7. Cardiovascular Outcomes by Cancer Treatment Class

We compared cardiovascular diagnoses recorded after cancer diagnosis among patients receiving specific cancer treatments to identify patterns according to baseline ECG ascertainment status.

Among patients treated with alkylating agents, composite CVD occurred in 90 of 766 patients (12%) with ECG vs. 39 of 156 (25%) without (*p* < 0.001), and HF in 42 (8.1%) vs. 25 (20%) (*p* < 0.001). Among patients treated with antimetabolites, composite CVD occurred in 40 of 477 (8.4%) vs. 26 of 125 (21%) (*p* < 0.001), and HF in 19 (5.6%) vs. 17 (18%) (*p* < 0.001).

Among patients treated with antineoplastic antibodies, composite CVD occurred in 26 of 345 (7.5%) vs. 16 of 55 (29%) (*p* < 0.001), HF in 16 (6.5%) vs. 11 (23%) (*p* = 0.002), and AFIB in seven (2.4%) vs. six (11%) (*p* = 0.012). Among those treated with hormonal therapies, composite CVD occurred in 50 of 449 (11%) vs. 31 of 101 (31%) (*p* < 0.001), HF in 24 (9.2%) vs. 20 (25%) (*p* < 0.001), and CAD in 13 (4.4%) vs. 11 (12%) (*p* = 0.026).

Among patients treated with chemotherapy rescue drugs, composite CVD was recorded in 15 of 181 (8.3%) vs. 11 of 43 (26%) (*p* = 0.018). Among patients treated with immune agents, HF was recorded in nine of 79 (11%) vs. 16 of 47 (34%) (*p* = 0.022). Among patients treated with mitotic inhibitors, composite CVD was recorded in 65 of 529 (12%) vs. 30 of 106 (28%) (*p* < 0.001), and HF in 29 (8.1%) vs. 23 (28%) (*p* < 0.001). Further details of treatment-specific outcomes are provided in Table 7, with outcome-specific denominators and NMiss counts in Supplemental Table S2.

### 3.8. Cardiovascular Outcomes by Sex and Race

The sex comparison included 1196 women and 1175 men. Overall CVD occurred in 14% of women and 15% of men (*p* = 0.8), HF in 12% vs. 11% (*p* = 0.9), AFIB in 5.9% vs. 8.2% (*p* = 0.4), CAD in 6.2% vs. 7.9% (*p* = 0.6), MI in <0.1% vs. 0.2% (*p* > 0.9), and stroke in 0.3% vs. 0.3% (*p* > 0.9). These subgroup comparisons are descriptive and exploratory.

The race comparison among patients treated with any treatment included 431 Black patients, 82 patients in the Other category, and 1858 White patients. Overall CVD was diagnosed in 16%, 13%, and 14%, respectively (*p* = 0.7). HF was diagnosed in 14%, 5.4%, and 11% (*p* = 0.5), and CAD in 8.6%, 7.4%, and 6.6% (*p* = 0.7). MI and stroke remained rare. The combined Other category is heterogeneous and does not support definitive subgroup-specific inference for Asian, Pacific Islander, or Native American participants.

## 4. Discussion

Our goal was to characterize historical real world baseline ECG ascertainment before cancer diagnosis and the linked cardiovascular information available within a large cancer survivor cohort. Baseline ECGs were available for most patients, and ECG ascertainment was associated with greater documented cardiovascular and cardiometabolic burden before cancer diagnosis. This pattern suggests that baseline ECG ascertainment may have functioned partly as a marker of greater pre-existing cardiovascular complexity, more intensive cardiovascular evaluation, or heightened clinician concern before cancer diagnosis. We refined the results by separating descriptive cardiovascular diagnosis frequencies after cancer diagnosis from newly documented diagnoses among patients without the corresponding prior documented diagnosis. The newly documented diagnosis analysis preserved the direction of the descriptive findings: overall CVD, HF, AFIB, CAD, and stroke were less frequent in the baseline ECG group, while MI remained rare and not significantly different. These findings are observational and do not establish that ECG testing itself reduced cardiovascular events. The principal contribution of the present analysis is therefore descriptive and epidemiologic: it characterizes who had a historical baseline ECG, how those patients differed before cancer diagnosis, and how subsequent documented cardiovascular diagnosis patterns differed between ascertainment groups. Accordingly, the specific question addressed by this study is whether historical pre-diagnostic ECG ascertainment within a clinically selected cancer survivor cohort identifies groups with different baseline cardiovascular burden and subsequent documented cardiovascular diagnosis patterns. The observed differences may reflect several overlapping clinical processes, including confounding by indication, variation in cardiovascular evaluation and healthcare engagement, oncologic heterogeneity, and changes in oncology and cardiovascular practice across 2000–2020; these factors frame the present findings as epidemiologic and hypothesis-generating and help define the variables and endpoint validation required for subsequent prospective and time to event investigation.

Cardiotoxic effects of cancer therapies are well established, with agents such as anthracyclines, HER2-targeted therapies, and radiation contributing to arrhythmias, myocarditis, ischemia, and heart failure [4,5]. Contemporary cardio-oncology recommendations increasingly emphasize comprehensive baseline cardiovascular risk assessment and optimization prior to potentially cardiotoxic therapies, including clinical history, physical examination, blood pressure assessment, laboratory evaluation, electrocardiography, biomarkers, imaging, lifestyle counseling, and exercise guidance when clinically appropriate [10]. Accordingly, the present study should not be interpreted as suggesting that resting ECG alone is sufficient for cardiovascular evaluation in patients with cancer. Importantly, this cohort spans historical practice eras both preceding and overlapping the emergence of modern cardio-oncology standardization. However, a standardized strategy for ECG utilization in broader cardio-oncology preventive care does not yet exist. Our findings provide evidence that baseline ECG acquisition—prior to cancer diagnosis and independent of treatment decision-making—may be associated with lower recorded frequencies of cardiovascular diagnoses. Among patients treated with cardiotoxic therapies such as anthracyclines, those who received a baseline ECG demonstrated lower recorded frequencies of HF and composite CVD compared to those who did not. These findings may inform future prospective investigations and implementation research on managing cardiotoxic therapies in cancer survivors and inform strategies for prevention of adverse cardiovascular outcomes. The present analysis characterizes ascertainment during 2000–2020, preceding the 2022 ESC recommendations. It does not test the effectiveness of a screening strategy. Because cardiovascular practice, oncology treatment, diagnostic coding, referral behavior, and the availability of cardio-oncology services evolved across this interval, clinical practice over time may have influenced both ECG ascertainment and subsequent diagnosis patterns. Oncology treatment, cardiovascular practice, coding, and cardio-oncology care evolved during the study period, and calendar time may therefore contribute residual temporal confounding.

Baseline ECG ascertainment prior to cancer diagnosis represents a measurable component of cardiovascular assessment within routine oncology practice. ECG availability may also function as a marker of broader care intensity, cardiovascular evaluation, surveillance, referral behavior, healthcare engagement, or clinician perception of cardiovascular risk. The substantially greater pre-existing cardiovascular and cardiometabolic burden among patients with baseline ECG ascertainment supports the possibility of confounding by indication. The adjusted model reduces but does not eliminate this concern, and no causal interpretation is made.

Shared risk factors between cancer and cardiovascular disease—including age, smoking, obesity, physical inactivity, diet, and genetic predisposition—may contribute to the observed associations. These shared factors may influence both the likelihood of receiving a baseline ECG and the risk of developing cardiovascular disease. In addition, ECG receipt may be associated with specific cancer diagnoses or treatment plans, reflecting clinician perception of cardiovascular risk. While ECG screening is not currently recommended for lower-risk populations by the United States Preventive Services Task Force or the European Society of Cardiology due to insufficient evidence of benefit [21,22], our findings support further investigation of cardiovascular assessment implementation strategies within higher-risk oncology populations. Clinically, these findings support examining how baseline ECG ascertainment may identify patients already embedded in more intensive cardiovascular evaluation pathways and how that information can be integrated with comorbidities, treatment exposures, imaging, and validated outcome timing in future studies.

Prior studies have examined the prognostic value of baseline ECG findings in oncology populations. An ECG score was evaluated for predicting cardiotoxicity in 134 therapy-naïve cancer patients [23], while baseline electrocardiographic and echocardiographic findings were examined for their associations with survival in patients with lung cancer [24].

The broader research program provides context for why historical ECG ascertainment and linkage are relevant. Prior completed work established the interdisciplinary cardio-oncology research infrastructure and cancer survivor cohort [25], demonstrated natural language processing extraction of ejection fraction and strain from cardiovascular imaging reports [26], and developed PrevCardioOncAI machine learning models for cardiovascular disease prediction in cancer survivors [27]. No artificial intelligence model was developed or tested in the present ECG ascertainment study. Within this broader research trajectory, the present study serves a distinct epidemiologic role by defining the historical availability, linkage, and clinical context of baseline ECG data needed to support subsequent endpoint validated cardiovascular analyses.

The present manuscript reports historical ECG ascertainment, linkage, descriptive cardiovascular diagnosis frequencies, newly documented diagnoses among patients without the corresponding prior documented diagnosis, treatment and demographic subgroup analyses, available follow up comparison, and an exploratory adjusted model of newly documented overall CVD diagnosis. Kaplan–Meier, Cox, competing risk, and machine learning survival prediction analyses were not performed in this study.

**Figure 6:**
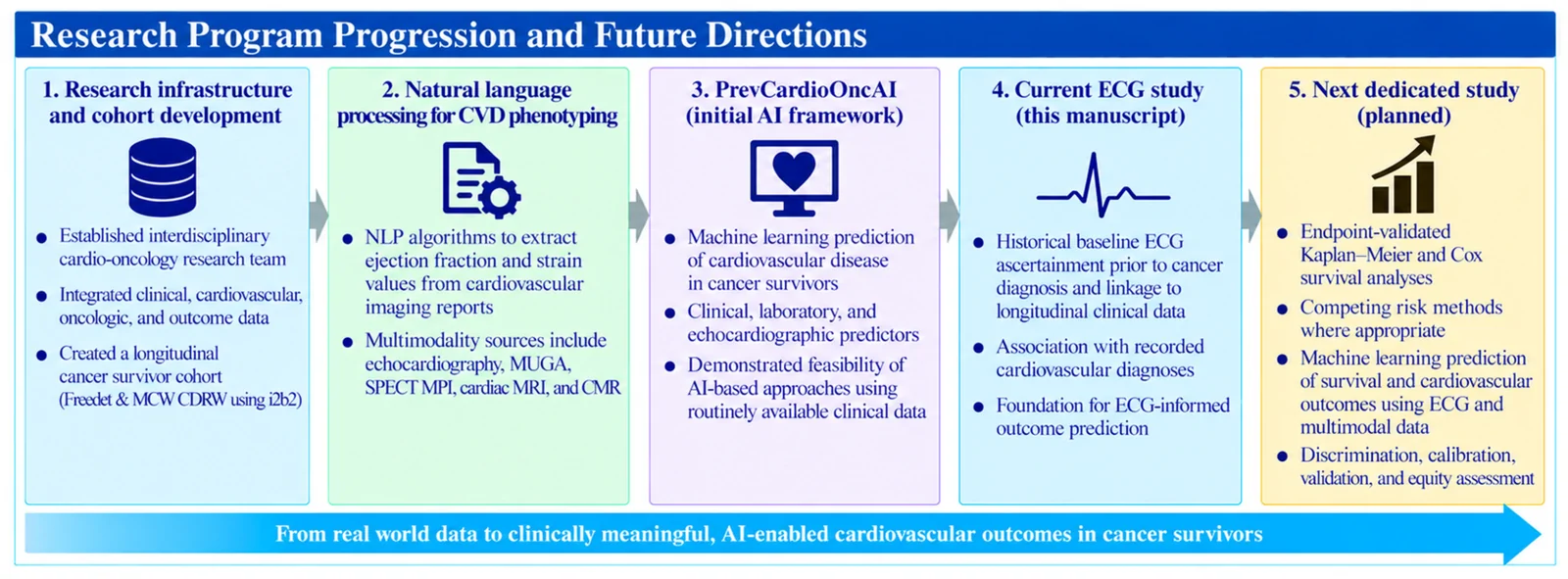
Research program progression and future directions. The first three stages correspond to prior publications establishing the interdisciplinary research infrastructure and cohort (Panel 1) [25], natural language processing extraction of cardiovascular imaging measurements (Panel 2) [26], and PrevCardioOncAI machine learning prediction of cardiovascular disease (Panel 3) [27]. The fourth stage represents the current ECG ascertainment study, and the fifth stage summarizes the planned endpoint validated survival and cardiovascular outcome prediction study.

The present study follows a sequence of prior completed work within this research program. Research infrastructure and cohort development preceded natural language processing extraction from cardiovascular imaging reports and PrevCardioOncAI machine learning prediction. The current study adds historical baseline ECG ascertainment, data linkage, descriptive post-cancer diagnosis frequencies, and newly documented cardiovascular diagnoses among patients without the corresponding prior documented diagnosis. A subsequent dedicated study is planned for endpoint validated time to event analysis and ECG-informed prediction after the required endpoint curation (Figure 6).

The current ECG analytic cohort was derived from the previously established cancer survivor resource. In the published parent cohort of 4626 cancer survivors, the most frequently represented cancer categories were hematologic cancers (744, 16%), lung cancer (711, 16%), breast cancer (502, 11%), and prostate cancer (401, 9%) [25]. These published values characterize the parent cohort and are not presented as ECG-specific subgroup counts because the current ECG analytic cohort contains 4592 participants. Cancer diagnosis information is available in the research resource, but cancer type distribution by baseline ECG status was not analyzed in the current study.

Published literature describes sex and gender disparities in cardiovascular care [28,29] and healthcare utilization among older adults [30]. Racial and ethnic disparities have also been described in cardiovascular disease among patients with cancer [31], preventive service utilization among older adults [32], receipt of cardiologist care during intensive care unit admission for heart failure [33], and cardiovascular disease burden and outcomes among African Americans [34].

From an equity perspective, baseline ECG ascertainment did not differ significantly between Black and White participants, while participants categorized as Other in the race variable had lower ECG ascertainment. The Other category combines heterogeneous populations, including Asian, Pacific Islander, and Native American participants, and the available subgroup output does not support reliable condition-specific inference within each of those populations. Individual level socioeconomic measures were not available for the current analysis. These limitations are stated explicitly, and the race findings are treated as descriptive rather than as genetic or biologic explanations of cardiovascular risk.

ECG is a widely available and comparatively low-cost cardiovascular test. AI-enabled ECG approaches have demonstrated the ability to identify left ventricular systolic dysfunction [35], and a pragmatic randomized trial demonstrated increased identification of low ejection fraction when AI-ECG results were available to clinicians [36]. Economic and implementation questions remain relevant to future prospective work, particularly if ECG-derived information is combined with validated predictive approaches. In a decision analytic model of AI-enabled ECG screening for asymptomatic left ventricular dysfunction, estimated costs were $43,351 per quality-adjusted life year gained for screening at age 65, with corresponding estimates of $48,649 at age 55 and $52,072 at age 75 [37]. The present retrospective study, however, was designed to characterize ascertainment and linked clinical data rather than to establish the cost effectiveness or clinical effectiveness of an ECG screening strategy.

Several limitations should be considered. The study was conducted within a single Wisconsin health system, and regional population characteristics and care delivery patterns may limit generalizability. Baseline ECG ascertainment was clinically selected rather than randomized, and patients with ECGs had substantially greater cardiovascular and cardiometabolic burden, creating confounding by indication and residual selection bias. Accordingly, the observed associations are most directly interpretable within a population of cancer survivors who had varying levels of prior cardiovascular evaluation, rather than as estimates applicable to an unselected cancer population. ECG availability may therefore reflect broader care intensity or healthcare engagement. The descriptive post-cancer diagnosis analyses contain outcome-specific missing values; Supplemental Table S2 reports the observed denominators and NMiss counts. The newly documented diagnosis analyses use the study variables as defined, with pr_* = no as the corresponding denominator and dn_* = yes as the newly documented diagnosis numerator. Available follow up distributions were similar between groups at the cohort level, but no standardized endpoint-specific observation window, validated first event time, censoring definition, or competing event framework was available for definitive Kaplan–Meier or Cox analysis. Cancer type information exists in the broader research resource, but cancer type distribution by baseline ECG status was not among the current analyses; tumor stage, tumor burden, progression, and cancer-specific mortality were not analyzed as current endpoints. Detailed antineoplastic treatment exposure was available, whereas longitudinal cardiovascular medication initiation, dose, duration, adherence, lifestyle factors, individual socioeconomic measures, and regular cardiology follow up were not uniformly analyzed across the retrospective period. The study spans 2000–2020, during which oncology treatment, cardiovascular practice, coding, and cardio-oncology care evolved; calendar time therefore remains a potential source of temporal confounding. The Other race category is heterogeneous, and sparse demographic and treatment subgroups limit definitive negative inference. These limitations preclude causal interpretation and support prospective and endpoint validated follow up studies.

Future work will address questions not analyzed in the current study. A dedicated endpoint validation phase will establish first event dates, time origins, censoring, competing events, and standardized at risk definitions before Kaplan–Meier, Cox, competing risk, and machine learning survival analyses. Future analyses will also incorporate calendar year; evaluate cancer type within the current ECG cohort; and incorporate tumor stage, disease burden, progression, cause specific mortality, cardiovascular medications, socioeconomic and healthcare access measures, and cardiology utilization where available. These future analyses are distinct from the completed analyses reported in the present manuscript.

## 5. Conclusions

Baseline ECGs were ascertainable before cancer diagnosis for most participants in this established cancer survivor cohort and could be linked with demographic characteristics, cancer treatment exposures, cardiovascular diagnoses, and longitudinal follow up. Patients with baseline ECG ascertainment had greater documented cardiovascular burden before cancer diagnosis. Descriptive cardiovascular diagnosis frequencies after cancer diagnosis were lower in the baseline ECG group, and the newly documented diagnosis analysis likewise showed lower newly documented overall CVD, HF, AFIB, CAD, and stroke diagnoses among patients without the corresponding prior documented diagnosis, while MI remained rare and not significantly different. These observational associations do not establish that ECG testing itself prevented cardiovascular events.

The findings support further prospective and endpoint validated investigation of baseline cardiovascular assessment in oncology populations. They also identify a large ECG-linked epidemiologic resource for subsequent time to event analyses after dedicated endpoint validation.

The present manuscript reports the completed historical ECG ascertainment and outcome characterization phase of the research program. Future work will address endpoint validated survival analysis and prediction after the required curation and validation.

## Figures and Tables

**Table 1:** Demographic data of individuals based on receipt of baseline ECG.

		No Baseline ECG	Baseline ECG	*p* Value (Significance = *p* < 0.05)
Overall Receipt n (%)		866 (19%)	3726 (81%)	N/A
Age n (SD)		66.94 (14.10)	69.17 (12.87)	*p* ≤ 0.001
Sex n (%)	Female	443 (51%)	1820 (49%)	*p* = 0.4
	Male	423 (49%)	1906 (51%)	
Race n (%)	White	665 (77%)	2912 (78%)	*p* = 0.14 between White and Black cancer survivors
	Black	135 (16%)	689 (18%)	
	Other (Asian, Pacific Islander, Native American)	66 (7.6%)	125 (3.4%)	*p* ≤ 0.001 between White and Other cancer survivors

**Table 2:** Epidemiologic data of individuals based on receipt of baseline ECG.

Outcome	Status	No Baseline ECG	Baseline ECG	*p* Value
Composite CVD Prior to Cancer Diagnosis n (%)	Not Diagnosed	544 (63%)	1774 (48%)	*p* ≤ 0.001
	Diagnosed	322 (37%)	1952 (52%)	
AFIB Prior to Cancer Diagnosis n (%)	Not Diagnosed	756 (87%)	2815 (76%)	*p* ≤ 0.001
	Diagnosed	110 (13%)	911 (24%)	
CAD Prior to Cancer Diagnosis n (%)	Not Diagnosed	631 (73%)	1935 (52%)	*p* ≤ 0.001
	Diagnosed	235 (27%)	1791 (48%)	
Cardiomegaly Prior to Cancer Diagnosis n (%)	Not Diagnosed	561 (65%)	1453 (39%)	*p* ≤ 0.001
	Diagnosed	305 (35%)	2273 (61%)	
Cardiomyopathy Prior to Cancer Diagnosis n (%)	Not Diagnosed	803 (93%)	3105 (83%)	*p* ≤ 0.001
	Diagnosed	63 (7.3%)	621 (17%)	
Restrictive Cardiomyopathy Prior to Cancer Diagnosis n (%)	Not Diagnosed	809 (93%)	3328 (89%)	*p* ≤ 0.001
	Diagnosed	57 (6.6%)	398 (11%)	
Cerebral Atherosclerosis Prior to Cancer Diagnosis n (%)	Not Diagnosed	861 (99%)	3659 (98%)	*p* = 0.029
	Diagnosed	5 (0.6%)	67 (1.8%)	
HF Prior to Cancer Diagnosis n (%)	Not Diagnosed	583 (67%)	1773 (48%)	*p* ≤ 0.001
	Diagnosed	283 (33%)	1953 (52%)	
MI Prior to Cancer Diagnosis n (%)	Not Diagnosed	815 (94%)	3369 (90%)	*p* = 0.002
	Diagnosed	51 (5.9%)	357 (9.6%)	
PAD Prior to Cancer Diagnosis n (%)	Not Diagnosed	738 (85%)	2598 (70%)	*p* ≤ 0.001
	Diagnosed	128 (15%)	1128 (30%)	
Stroke Prior to Cancer Diagnosis n (%)	Not Diagnosed	739 (85%)	2937 (79%)	*p* ≤ 0.001
	Diagnosed	127 (15%)	789 (21%)	
UPVD Prior to Cancer Diagnosis n (%)	Not Diagnosed	796 (92%)	3150 (85%)	*p* ≤ 0.001
	Diagnosed	70 (8.1%)	576 (15%)	
Diabetes Prior to Cancer Diagnosis n (%)	Not Diagnosed	735 (85%)	2773 (74%)	*p* ≤ 0.001
	Diagnosed	131 (15%)	953 (26%)	
HTN Prior to Cancer Diagnosis n (%)	Not Diagnosed	368 (42%)	680 (18%)	*p* ≤ 0.001
	Diagnosed	498 (58%)	3046 (82%)	
HL Prior to Cancer Diagnosis n (%)	Not Diagnosed	561 (65%)	1258 (34%)	*p* ≤ 0.001
	Diagnosed	305 (35%)	2468 (66%)	
PE Prior to Cancer Diagnosis n (%)	Not Diagnosed	702 (81%)	2461 (66%)	*p* ≤ 0.001
	Diagnosed	164 (19%)	1265 (34%)	
Orthopnea Prior to Cancer Diagnosis n (%)	Not Diagnosed	862 (100%)	3663 (98%)	*p* = 0.022
	Diagnosed	4 (0.5%)	63 (1.7%)	
Shortness of Breath Prior to Cancer Diagnosis n (%)	Not Diagnosed	500 (58%)	1469 (39%)	*p* ≤ 0.001
	Diagnosed	366 (42%)	2257 (61%)	
Fatigue Prior to Cancer Diagnosis n (%)	Not Noted	639 (74%)	1918 (51%)	*p* ≤ 0.001
	Noted	227 (26%)	1808 (49%)	
Composite CVD After Cancer Diagnosis n (%)	Not Diagnosed	653 (75%)	3354 (90%)	*p* ≤ 0.001
	Diagnosed	213 (25%)	372 (10.0%)	
HF After Cancer Diagnosis n (%)	Not Diagnosed	475 (79%)	2069 (94%)	*p* ≤ 0.001
	Diagnosed	123 (21%)	137 (6.2%)	
AFIB After Cancer Diagnosis n (%)	Not Diagnosed	680 (90%)	2878 (95%)	*p* ≤ 0.001
	Diagnosed	77 (10%)	157 (5.2%)	
CAD After Cancer Diagnosis n (%)	Not Diagnosed	598 (89%)	2303 (95%)	*p* ≤ 0.001
	Diagnosed	74 (11%)	129 (5.3%)	
MI After Cancer Diagnosis n (%)	Not Diagnosed	864 (100%)	3698 (100%)	*p* = 0.7
	Diagnosed	2 (0.2%)	3 (<0.1%)	
Stroke After Cancer Diagnosis n (%)	Not Diagnosed	839 (98%)	3640 (100%)	*p* ≤ 0.001
	Diagnosed	15 (1.8%)	5 (0.1%)	

AFIB = atrial fibrillation, CAD = coronary artery disease, HF = heart failure, MI = myocardial infarction, PAD = peripheral arterial disease, UPVD = unspecified peripheral vascular disease, HTN = hypertension, HLP = hyperlipidemia, PE = peripheral edema, SOB = shortness of breath. Cardiovascular diagnoses documented before and after cancer diagnosis were tabulated separately. For the post-cancer diagnosis rows, percentages reproduce the observed yes/no outcome denominator from the analysis output; outcome-specific denominators and NMiss counts are provided in Supplemental Table S2.

**Table 4:** Available follow up duration according to baseline ECG ascertainment.

Measure	No Baseline ECG (*N* = 866)	Baseline ECG (*N* = 3726)	*p* Value
Survdays, median (Q1, Q3)	748 (258, 1781)	767 (219, 1822)	>0.9
Unknown, n	63	308	
Test	Wilcoxon rank-sum		

**Table 5:** Exploratory multivariable logistic regression for newly documented overall CVD diagnosis after cancer diagnosis.

Predictor	Odds Ratio	95% CI	*p* Value
Baseline ECG ascertainment	0.38	0.31–0.46	<0.001
Age	1.00	0.99–1.01	0.86
Male sex	1.09	0.92–1.31	0.33
Race: Other	0.67	0.40–1.09	0.12
Race: White	0.93	0.74–1.18	0.55
Comorbidity count	0.93	0.89–0.97	0.0004
Treatment count	1.30	0.06–9.53	0.82

Reference category for race: Black. The model evaluates odds of a newly documented overall CVD diagnosis after cancer diagnosis and does not estimate time-specific cardiovascular risk. First event dates, censoring, loss to follow up, and competing events are not incorporated in this logistic model.

**Table 7:** Cardiovascular diagnoses recorded after cancer diagnosis by cancer treatment type and baseline ECG ascertainment.

Treatment Type	CVD	Baseline ECG Receipt	Developed CVD	*p* Value
Alkylating Agents	Composite CVD	No Baseline ECG n (%)	39 (25%)	<0.001
		Baseline ECG n (%)	90 (12%)	
	HF	No Baseline ECG n (%)	25 (20%)	<0.001
		Baseline ECG n (%)	42 (8.1%)	
	AFIB	No Baseline ECG n (%)	15 (10%)	0.098
		Baseline ECG n (%)	36 (5.6%)	
	CAD	No Baseline ECG n (%)	9 (7%)	0.749
		Baseline ECG n (%)	29 (5.3%)	
	MI	No Baseline ECG n (%)	0 (0%)	-
		Baseline ECG n (%)	0 (0%)	
	Stroke	No Baseline ECG n (%)	1 (0.7%)	0.752
		Baseline ECG n (%)	1 (0.1%)	
Antimetabolites	Composite CVD	No Baseline ECG n (%)	26 (21%)	<0.001
		Baseline ECG n (%)	40 (8.4%)	
	HF	No Baseline ECG n (%)	17 (18%)	<0.001
		Baseline ECG n (%)	19 (5.6%)	
	AFIB	No Baseline ECG n (%)	9 (7.6%)	0.188
		Baseline ECG n (%)	14 (3.4%)	
	CAD	No Baseline ECG n (%)	5 (5%)	>0.999
		Baseline ECG n (%)	15 (4.3%)	
	MI	No Baseline ECG n (%)	1 (0.8%)	0.711
		Baseline ECG n (%)	0 (0%)	
	Stroke	No Baseline ECG n (%)	0 (0%)	>0.999
		Baseline ECG n (%)	2 (0.4%	
Angiogenesis Inhibitors	Composite CVD	No Baseline ECG n (%)	1 (9.1%)	>0.999
		Baseline ECG n (%)	11 (11%)	
	HF	No Baseline ECG n (%)	1 (14%)	>0.999
		Baseline ECG n (%)	7 (10%)	
	AFIB	No Baseline ECG n (%)	0 (0%)	>0.999
		Baseline ECG n (%)	6 (7.4%)	
	CAD	No Baseline ECG n (%)	0 (0%)	>0.999
		Baseline ECG n (%)	4 (5.6%)	
	MI	No Baseline ECG n (%)	0 (0%)	-
		Baseline ECG n (%)	0 (0%)	
	Stroke	No Baseline ECG n (%)	0 (0%)	-
		Baseline ECG n (%)	0 (0%)	
HER2 Antibodies	Composite CVD	No Baseline ECG n (%)	1 (14%)	>0.999
		Baseline ECG n (%)	7 (15%)	
	HF	No Baseline ECG n (%)	1 (14%)	>0.999
		Baseline ECG n (%)	5 (18%)	
	AFIB	No Baseline ECG n (%)	0 (0%)	-
		Baseline ECG n (%)	0 (0%)	
	CAD	No Baseline ECG n (%)	0 (0%)	>0.999
		Baseline ECG n (%)	1 (2.9%)	
	MI	No Baseline ECG n (%)	0 (0%)	-
		Baseline ECG n (%)	0 (0%)	
	Stroke	No Baseline ECG n (%)	0 (0%)	>0.999
		Baseline ECG n (%)	1 (2.2%)	
Antineoplastic Antibodies	Composite CVD	No Baseline ECG n (%)	16 (29%)	<0.001
		Baseline ECG n (%)	26 (7.5%)	
	HF	No Baseline ECG n (%)	11 (23%)	0.002
		Baseline ECG n (%)	16 (6.5%)	
	AFIB	No Baseline ECG n (%)	6 (11%)	0.012
		Baseline ECG n (%)	7 (2.4%)	
	CAD	No Baseline ECG n (%)	2 (4.5%)	>0.999
		Baseline ECG n (%)	7 (3%)	
	MI	No Baseline ECG n (%)	0 (0%)	-
		Baseline ECG n (%)	0 (0%)	
	Stroke	No Baseline ECG n (%)	0 (0%)	>0.999
		Baseline ECG n (%)	01 (0.3%)	
Antineoplastic Hormonal Drugs	Composite CVD	No Baseline ECG n (%)	31 (31%)	<0.001
		Baseline ECG n (%)	50 (11%)	
	HF	No Baseline ECG n (%)	20 (25%)	<0.001
		Baseline ECG n (%)	24 (9.2%)	
	AFIB	No Baseline ECG n (%)	9 (9.7%)	0.442
		Baseline ECG n (%)	22 (6%)	
	CAD	No Baseline ECG n (%)	11 (12%)	0.026
		Baseline ECG n (%)	13 (4.4%)	
	MI	No Baseline ECG n (%)	0 (0%)	>0.999
		Baseline ECG n (%)	1 (0.2%)	
	Stroke	No Baseline ECG n (%)	1 (1%)	0.967
		Baseline ECG n (%)	1 (0.2%)	
Antineoplastic Antibiotics	Composite CVD	No Baseline ECG n (%)	24 (22%)	0.157
		Baseline ECG n (%)	51 (15%)	
	HF	No Baseline ECG n (%)	15 (17%)	0.157
		Baseline ECG n (%)	21 (9.1%)	
	AFIB	No Baseline ECG n (%)	10 (9.8%)	0.574
		Baseline ECG n (%)	20 (6.8%)	
	CAD	No Baseline ECG n (%)	5 (5.3%)	0.634
		Baseline ECG n (%)	19 (7.5%)	
	MI	No Baseline ECG n (%)	0 (0%)	-
		Baseline ECG n (%)	0 (0%)	
	Stroke	No Baseline ECG n (%)	0 (0%)	-
		Baseline ECG n (%)	0 (0%)	
Combined Antineoplastic	Composite CVD	No Baseline ECG n (%)	0 (0%)	-
		Baseline ECG n (%)	0 (0%)	
	HF	No Baseline ECG n (%)	0 (0%)	-
		Baseline ECG n (%)	0 (0%)	
	AFIB	No Baseline ECG n (%)	0 (0%)	-
		Baseline ECG n (%)	0 (0%)	
	CAD	No Baseline ECG n (%)	0 (0%)	-
		Baseline ECG n (%)	0 (0%)	
	MI	No Baseline ECG n (%)	0 (0%)	-
		Baseline ECG n (%)	0 (0%)	
	Stroke	No Baseline ECG n (%)	0 (0%)	-
		Baseline ECG n (%)	0 (0%)	
Antineoplastic Enzymes	Composite CVD	No Baseline ECG n (%)	8 (18%)	0.960
		Baseline ECG n (%)	26 (13%)	
	HF	No Baseline ECG n (%)	5 (13%)	0.960
		Baseline ECG n (%)	12 (8.8%)	
	AFIB	No Baseline ECG n (%)	4 (9.3%)	0.960
		Baseline ECG n (%)	11 (6.5%)	
	CAD	No Baseline ECG n (%)	2 (4.9%)	>0.999
		Baseline ECG n (%)	8 (5.2%)	
	MI	No Baseline ECG n (%)	0 (0%)	-
		Baseline ECG n (%)	0 (0%)	
	Stroke	No Baseline ECG n (%)	1 (2.3%)	0.960
		Baseline ECG n (%)	0 (0%)	
Miscellaneous Antineoplastics	Composite CVD	No Baseline ECG n (%)	8 (22%)	0.603
		Baseline ECG n (%)	14 (13%)	
	HF	No Baseline ECG n (%)	5 (17%)	0.603
		Baseline ECG n (%)	7 (9.2%)	
	AFIB	No Baseline ECG n (%)	2 (5.6%)	>0.999
		Baseline ECG n (%)	4 (4.3%)	
	CAD	No Baseline ECG n (%)	1 (3%)	0.603
		Baseline ECG n (%)	7 (9.7%)	
	MI	No Baseline ECG n (%)	0 (0%)	-
		Baseline ECG n (%)	0 (0%)	
	Stroke	No Baseline ECG n (%)	0 (0%)	-
		Baseline ECG n (%)	0 (0%)	
Chemotherapy Rescue Drugs	Composite CVD	No Baseline ECG n (%)	11 (26%)	0.018
		Baseline ECG n (%)	15 (8.3%)	
	HF	No Baseline ECG n (%)	7 (20%)	0.086
		Baseline ECG n (%)	9 (6.6%)	
	AFIB	No Baseline ECG n (%)	4 (9.3%)	0.098
		Baseline ECG n (%)	3 (1.9%)	
	CAD	No Baseline ECG n (%)	2 (5.4%)	>0.999
		Baseline ECG n (%)	7 (5.1%)	
	MI	No Baseline ECG n (%)	0 (0%)	-
		Baseline ECG n (%)	0 (0%)	
	Stroke	No Baseline ECG n (%)	0 (0%)	>0.999
		Baseline ECG n (%)	1 (0.6%)	
Immune Agents	Composite CVD	No Baseline ECG n (%)	17 (32%)	0.355
		Baseline ECG n (%)	24 (20%)	
	HF	No Baseline ECG n (%)	16 (34%)	0.022
		Baseline ECG n (%)	9 (11%)	
	AFIB	No Baseline ECG n (%)	7 (13%)	>0.999
		Baseline ECG n (%)	10 (9.8%)	
	CAD	No Baseline ECG n (%)	4 (8.9%)	>0.999
		Baseline ECG n (%)	8 (8.7%)	
	MI	No Baseline ECG n (%)	0 (0%)	>0.999
		Baseline ECG n (%)	1 (0.8%)	
	Stroke	No Baseline ECG n (%)	0 (0%)	-
		Baseline ECG n (%)	0 (0%)	
Mitotic Inhibitors	Composite CVD	No Baseline ECG n (%)	30 (28%)	<0.001
		Baseline ECG n (%)	65 (12%)	
	HF	No Baseline ECG n (%)	23 (28%)	<0.001
		Baseline ECG n (%)	29 (8.1%)	
	AFIB	No Baseline ECG n (%)	11 (11%)	0.117
		Baseline ECG n (%)	25 (5.5%)	
	CAD	No Baseline ECG n (%)	5 (5.9%)	>0.999
		Baseline ECG n (%)	24 (6.6%)	
	MI	No Baseline ECG n (%)	0 (0%)	-
		Baseline ECG n (%)	0 (0%)	
	Stroke	No Baseline ECG n (%)	0 (0%)	>0.999
		Baseline ECG n (%)	2 (0.4%)	

## Data Availability

The datasets presented in this article are not readily available due to technical/time limitations. Requests to access the datasets should be directed to Sherry-Ann Brown.

## Supplementary Materials

The following supporting information can be downloaded at https://www.mdpi.com/article/10.3390/cancers18203306/s1, Figure S1: Multimodal data linkage and data sources for the historical baseline ECG cohort; Table S1: The antineoplastic classes and associated drugs defined by Medispan that were included in this study; Table S2: Cardiovascular diagnoses after cancer diagnosis: denominators and missing counts overall and by medication category.
