# Supplementary material for: Historical Baseline Electrocardiogram Ascertainment Prior to Cancer Diagnosis and Cardiovascular Diagnosis Patterns in Cancer Survivors: A Retrospective Observational Cohort Study
Source: Cancers (Basel). 2026 Oct 14;18(20):3306. doi: 10.3390/cancers18203306 (PMC13702632; doi:10.3390/cancers18203306)
Supplement: Supplementary file 1 [file cancers-18-03306-s001.zip › cancers-4396567-supplementary.pdf]

Supplemental Material

Table S1. Antineoplastic classes and associated drugs defined by Medispan that were included in this study.

Drug Class	Drugs Included
**Alkylating Agents**	bendamustine, busulfan, carboplatin, carmustine, chlorambucil, cisplatin, cyclophosphamide, ifosfamide, lomustine, lurbinectedin, mechlorethamine, melphalan, oxaliplatin, streptozocin, temozolomide, thiotepa, trabectedin
**Antimetabolites**	azacitidine, capecitabine, cladribine, clofarabine, cytarabine, decitabine, floxuridine, fludarabine, fluorouracil, gemcitabine, mercaptopurine, methotrexate, nelarabine, pemetrexed, pralatrexate, thioguanine
**Antineoplastic Angiogenesis inhibitors**	aflibercept, axitinib, bevacizumab, lenvatinib, ramucirumab
**Anti HER Antibodies**	margetuximab, pertuzumab, trastuzumab, tucatinib
**Antineoplastic Antibodies**	alemtuzumab, atezolizumab, avelumab, belantamab mafodotin, blinatumomab, brentuximab vedotin, cemiplimab, daratumumab, durvalumab, elotuzumab, enfortumab, gemtuzumab ozogamicin, inotuzumab ozogamicin, ipilimumab, isatuximab, mogamulizumab, nivolumab, obinutuzumab, ofatumumab, panitumumab, pembrolizumab, polatuzumab vedotin, rituximab, tafasitamab, trastuzumab
**Antineoplastic Hormonal Drugs**	abiraterone, anastrozole, apalutamide, bicalutamide, darolutamide, degarelix, enzalutamide, exemestane, flutamide, fulvestrant, goserelin, histrelin, letrozole, leuprolide, medroxyprogesterone, megestrol, mitotane, nilutamide, relugolix, tamoxifen, toremifene, triptorelin
**Antineoplastic Antibiotics (anthracyclines)**	bleomycin, dactinomycin, daunorubicin, doxorubicin, epirubicin, idarubicin, mitomycin, mitoxantrone, valrubicin
**Combined Antineoplastics**	cytarabine, daratumumab, daunorubicin, hyaluronidase, nivolumab, pertuzumab, tipiracil, trastuzumab, trifluridine
**Antineoplastic Enzyme Inhibitors**	abemaciclib, acalabrutinib, alectinib, alpelisib, belinostat, binimetinib, bortezomib, bosutinib, cabozantinib, cabozantinib, capmatinib, carfilzomib, ceritinib, copanlisib, crizotinib, dabrafenib, dasatinib, duvelisib, enasidenib, encorafenib, entrectinib, erdafitinib, everolimus, fedratinib, gilteritinib, ibrutinib, idelalisib, imatinib, imatinib, ivosidenib, ixazomib, lapatinib, larotrectinib, lorlatinib, midostaurin, neratinib, nilotinib, niraparib, olaparib, palbociclib, panobinostat, pazopanib, ponatinib, pralsetinib, regorafenib, ribociclib, ripretinib, romidepsin, rucaparib, ruxolitinib, selpercatinib, sirolimus, sorafenib, sunitinib, tazemetostat, temsirolimus, trametinib, vemurafenib, vorinostat, zanubrutinib, asparaginase, pegaspargase

**Miscellaneous Antineoplastics**	aldesleukin, arsenic trioxide, BCG, live, Tice strain, bexarotene, dacarbazine, denileukin diftitox, dihematoporphyrin ether, hydroxyurea, interferon alfa-2a, interferon alfa-2b, interferon gamma-1b, methoxsalen, omacetaxine mepesuccinate, pentostatin, procarbazine, tagraxofusp, tretinoin
**Chemo Rescue Drugs**	amifostine, dexrazoxane, glucarpidase, leucovorin, levoleucovorin, mesna
**Immune Agents**	axicabtagene ciloleucel, brexucabtagene autoleucel, sipuleucel-T, tisagenlecleucel
**Mitotic Inhibitors**	cabazitaxel, docetaxel, eribulin, etoposide, ixabepilone, paclitaxel, vinblastine, vincristine, vinorelbine

Table S2. Cardiovascular diagnoses after cancer diagnosis: denominators and missing counts overall and by medication category. Overall cohort and the 13 medication categories reported in manuscript Table 7. Values reproduce the analysis output, with the observed denominator for each diagnosis shown explicitly.

p value	Baseline ECG		No baseline ECG		Outcome
	NMiss	n/N (%)	NMiss	n/N (%)	
**Overall cohort Group totals: 866 without baseline ECG; 3,726 with baseline ECG.**					
<0.001	0†	372/3,726 (10.0%)	0†	213/866 (25%)	Composite CVD
<0.001	1,520	137/2,206 (6.2%)	268	123/598 (21%)	HF
<0.001	691	157/3,035 (5.2%)	109	77/757 (10%)	AFIB
<0.001	1,294	129/2,432 (5.3%)	194	74/672 (11%)	CAD
0.7	25	3/3,701 (<0.1%)	0	2/866 (0.2%)	MI
<0.001	81	5/3,645 (0.1%)	12	15/854 (1.8%)	Stroke
**Alkylating agents Group totals: 156 without baseline ECG; 766 with baseline ECG.**					
<0.001	0†	90/766 (12%)	0†	39/156 (25%)	Composite CVD
<0.001	245	42/521 (8.1%)	33	25/123 (20%)	HF
0.098	118	36/648 (5.6%)	9	15/147 (10%)	AFIB
0.749	220	29/546 (5.3%)	27	9/129 (7.0%)	CAD
—	2	0/764 (0%)	0	0/156 (0%)	MI
0.752	11	1/755 (0.1%)	5	1/151 (0.7%)	Stroke
**Antimetabolites Group totals: 125 without baseline ECG; 477 with baseline ECG.**					
<0.001	0†	40/477 (8.4%)	0†	26/125 (21%)	Composite CVD
<0.001	140	19/337 (5.6%)	30	17/95 (18%)	HF
0.188	71	14/406 (3.4%)	6	9/119 (7.6%)	AFIB
>0.999	132	15/345 (4.3%)	24	5/101 (5.0%)	CAD
0.711	4	0/473 (0%)	0	1/125 (0.8%)	MI
>0.999	9	2/468 (0.4%)	0	0/125 (0%)	Stroke
**Angiogenesis inhibitors Group totals: 11 without baseline ECG; 99 with baseline ECG.**					
>0.999	0†	11/99 (11%)	0†	1/11 (9.1%)	Composite CVD
>0.999	32	7/67 (10%)	4	1/7 (14%)	HF
>0.999	18	6/81 (7.4%)	0	0/11 (0%)	AFIB
>0.999	27	4/72 (5.6%)	2	0/9 (0%)	CAD
—	0†	0/99 (0%)	0†	0/11 (0%)	MI
—	2	0/97 (0%)	0	0/11 (0%)	Stroke
**HER2 antibodies Group totals: 7 without baseline ECG; 48 with baseline ECG.**					
>0.999	0†	7/48 (15%)	0†	1/7 (14%)	Composite CVD
>0.999	20	5/28 (18%)	0	1/7 (14%)	HF

p value	Baseline ECG		No baseline ECG		Outcome
	NMiss	n/N (%)	NMiss	n/N (%)	
—	8	0/40 (0%)	0	0/7 (0%)	AFIB
>0.999	14	1/34 (2.9%)	2	0/5 (0%)	CAD
—	1	0/47 (0%)	0	0/7 (0%)	MI
>0.999	2	1/46 (2.2%)	0	0/7 (0%)	Stroke
**Antineoplastic antibodies Group totals: 55 without baseline ECG; 345 with baseline ECG.**					
<0.001	0†	26/345 (7.5%)	0†	16/55 (29%)	Composite CVD
0.002	98	16/247 (6.5%)	7	11/48 (23%)	HF
0.012	56	7/289 (2.4%)	1	6/54 (11%)	AFIB
>0.999	115	7/230 (3.0%)	11	2/44 (4.5%)	CAD
—	0†	0/345 (0%)	0†	0/55 (0%)	MI
>0.999	3	1/342 (0.3%)	1	0/54 (0%)	Stroke
**Antineoplastic hormonal drugs Group totals: 101 without baseline ECG; 449 with baseline ECG.**					
<0.001	0†	50/449 (11%)	0†	31/101 (31%)	Composite CVD
0.001	189	24/260 (9.2%)	21	20/80 (25%)	HF
0.442	80	22/369 (6.0%)	8	9/93 (9.7%)	AFIB
0.026	152	13/297 (4.4%)	12	11/89 (12%)	CAD
>0.999	3	1/446 (0.2%)	0	0/101 (0%)	MI
0.967	11	1/438 (0.2%)	3	1/98 (1.0%)	Stroke
**Antineoplastic antibiotics Group totals: 107 without baseline ECG; 349 with baseline ECG.**					
0.157	0†	51/349 (15%)	0†	24/107 (22%)	Composite CVD
0.157	119	21/230 (9.1%)	21	15/86 (17%)	HF
0.574	53	20/296 (6.8%)	5	10/102 (9.8%)	AFIB
0.634	96	19/253 (7.5%)	13	5/94 (5.3%)	CAD
—	1	0/348 (0%)	0	0/107 (0%)	MI
—	6	0/343 (0%)	0	0/107 (0%)	Stroke
**Combined antineoplastics Group totals: 3 without baseline ECG; 25 with baseline ECG.**					
—	0†	0/25 (0%)	0†	0/3 (0%)	Composite CVD
—	9	0/16 (0%)	0	0/3 (0%)	HF
—	3	0/22 (0%)	0	0/3 (0%)	AFIB
—	10	0/15 (0%)	0	0/3 (0%)	CAD
—	0†	0/25 (0%)	0†	0/3 (0%)	MI
—	0	0/25 (0%)	0	0/3 (0%)	Stroke
**Antineoplastic enzyme inhibitors Group totals: 44 without baseline ECG; 201 with baseline ECG.**					
0.960	0†	26/201 (13%)	0†	8/44 (18%)	Composite CVD
0.960	65	12/136 (8.8%)	6	5/38 (13%)	HF
0.960	33	11/168 (6.5%)	1	4/43 (9.3%)	AFIB
>0.999	46	8/155 (5.2%)	3	2/41 (4.9%)	CAD
—	2	0/199 (0%)	0	0/44 (0%)	MI
0.960	1	0/200 (0%)	0	1/44 (2.3%)	Stroke
**Miscellaneous antineoplastics Group totals: 36 without baseline ECG; 110 with baseline ECG.**					
0.603	0†	14/110 (13%)	0†	8/36 (22%)	Composite CVD
0.603	34	7/76 (9.2%)	6	5/30 (17%)	HF
>0.999	17	4/93 (4.3%)	0	2/36 (5.6%)	AFIB
0.603	38	7/72 (9.7%)	3	1/33 (3.0%)	CAD
—	0†	0/110 (0%)	0†	0/36 (0%)	MI
—	1	0/109 (0%)	0	0/36 (0%)	Stroke
**Chemotherapy rescue drugs Group totals: 43 without baseline ECG; 181 with baseline ECG.**					
0.018	0†	15/181 (8.3%)	0†	11/43 (26%)	Composite CVD
0.086	44	9/137 (6.6%)	8	7/35 (20%)	HF
0.098	22	3/159 (1.9%)	0	4/43 (9.3%)	AFIB

p value	Baseline ECG		No baseline ECG		Outcome
	NMiss	n/N (%)	NMiss	n/N (%)	
>0.999	43	7/138 (5.1%)	6	2/37 (5.4%)	CAD
—	2	0/179 (0%)	0	0/43 (0%)	MI
>0.999	4	1/177 (0.6%)	0	0/43 (0%)	Stroke
**Immune agents Group totals: 53 without baseline ECG; 118 with baseline ECG.**					
0.355	0†	24/118 (20%)	0†	17/53 (32%)	Composite CVD
0.022	39	9/79 (11%)	6	16/47 (34%)	HF
>0.999	16	10/102 (9.8%)	1	7/52 (13%)	AFIB
>0.999	26	8/92 (8.7%)	8	4/45 (8.9%)	CAD
>0.999	0†	1/118 (0.8%)	0†	0/53 (0%)	MI
—	0	0/118 (0%)	1	0/52 (0%)	Stroke
**Mitotic inhibitors Group totals: 106 without baseline ECG; 529 with baseline ECG.**					
<0.001	0†	65/529 (12%)	0†	30/106 (28%)	Composite CVD
<0.001	171	29/358 (8.1%)	23	23/83 (28%)	HF
0.117	77	25/452 (5.5%)	7	11/99 (11%)	AFIB
>0.999	163	24/366 (6.6%)	21	5/85 (5.9%)	CAD
—	2	0/527 (0%)	0	0/106 (0%)	MI
>0.999	12	2/517 (0.4%)	3	0/103 (0%)	Stroke

Definitions. n is the number coded yes for the corresponding diagnosis; N is the sum of counts coded yes and no for that outcome within the relevant ECG group. NMiss reproduces the count labeled missing in the analysis output. Interpretation. Supplemental Table S2 supports the descriptive post cancer diagnosis analyses in Table 2 and Table 7. It is separate from the newly documented diagnosis analyses in Table 3 and Figure 4. Source. Revised_08072024 CVOutcomes in Cancer Survivors - Analysis Results(1).xlsx; overall cohort from worksheet Table 1a and medication categories from worksheets Table 2a and Table 2b.

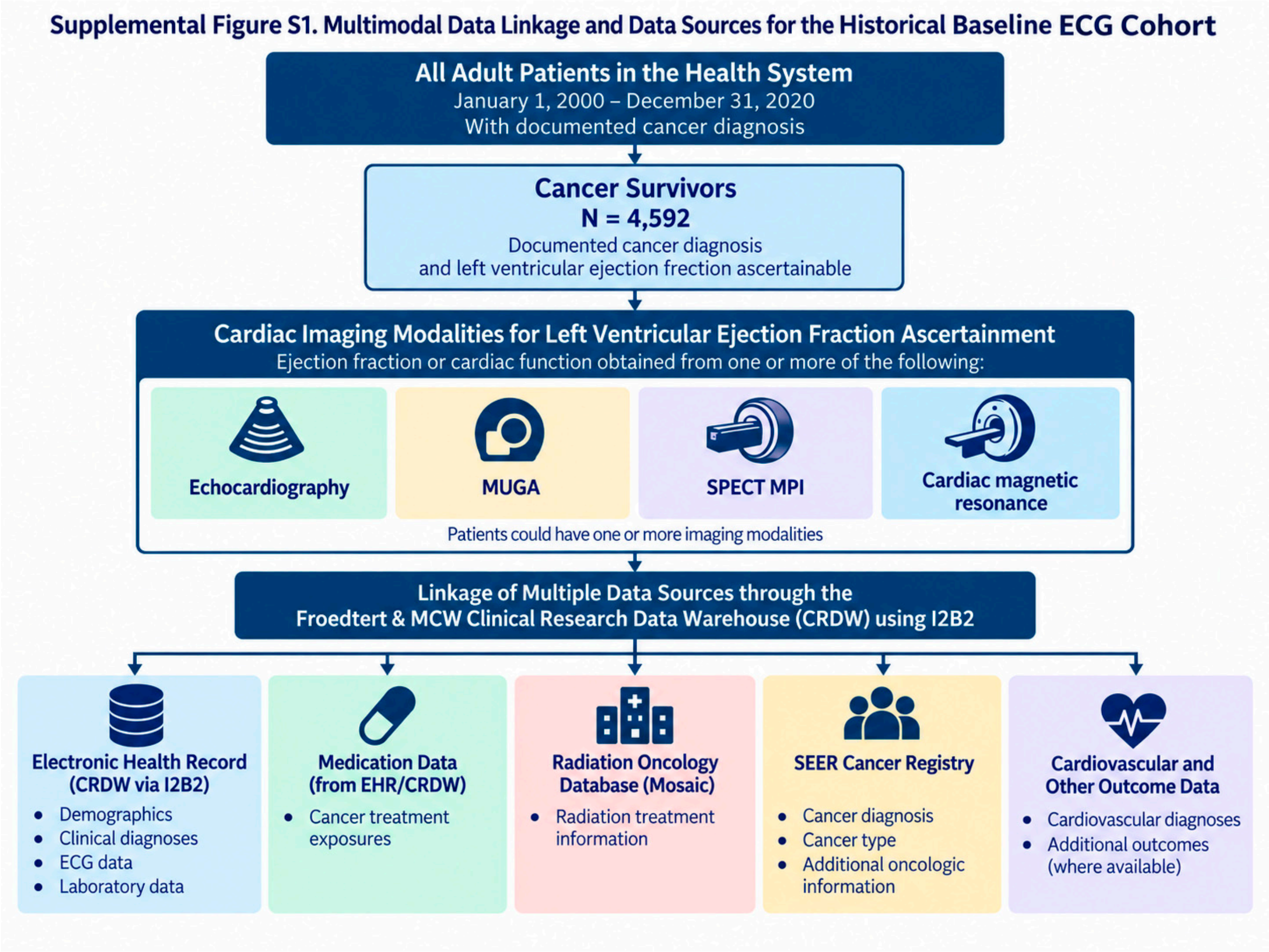

Figure S1. Multimodal data linkage and data sources for the historical baseline ECG cohort. The figure summarizes the cancer survivor cohort, multimodality cardiac function ascertainment, CRDW/I2B2 linkage, medication exposure, Mosaic radiation oncology information, SEER cancer registry information, and cardiovascular outcome data.
